# Functional Characterization of *Corynebacterium glutamicum* Mycothiol S-Conjugate Amidase

**DOI:** 10.1371/journal.pone.0115075

**Published:** 2014-12-16

**Authors:** Meiru Si, Mingxiu Long, Muhammad Tausif Chaudhry, Yixiang Xu, Pan Zhang, Lei Zhang, Xihui Shen

**Affiliations:** 1 State Key Laboratory of Crop Stress Biology for Arid Areas and College of Life Sciences, Northwest A&F University, Yangling, Shaanxi, China; 2 College of Animal Science and Technology, Northwest A&F University, Yangling, Shaanxi, China; 3 Environmental Analytical Laboratory, National Physical & Standards Laboratory, PCSIR, Islamabad, Pakistan; Beijing Institute of Microbiology and Epidemiology, China

## Abstract

The present study focuses on the genetic and biochemical characterization of mycothiol *S*-conjugate amidase (Mca) of *Corynebacterium glutamicum*. Recombinant *C. glutamicum* Mca was heterologously expressed in *Escherichia coli* and purified to apparent homogeneity. The molecular weight of native Mca protein determined by gel filtration chromatography was 35 kDa, indicating that Mca exists as monomers in the purification condition. Mca showed amidase activity with mycothiol *S*-conjugate of monobromobimane (MSmB) *in vivo* while *mca* mutant lost the ability to cleave MSmB. In addition, Mca showed limited deacetylase activity with *N*-acetyl-D-glucosamine (GlcNAc) as substrate. Optimum pH for amidase activity was between 7.5 and 8.5, while the highest activity in the presence of Zn^2+^ confirmed Mca as a zinc metalloprotein. Amino acid residues conserved among Mca family members were located in *C. glutamicum* Mca and site-directed mutagenesis of these residues indicated that Asp14, Tyr137, His139 and Asp141 were important for activity. The *mca* deletion mutant showed decreased resistance to antibiotics, alkylating agents, oxidants and heavy metals, and these sensitive phenotypes were recovered in the complementary strain to a great extent. The physiological roles of Mca in resistance to various toxins were further supported by the induced expression of Mca in *C. glutamicum* under various stress conditions, directly under the control of the stress-responsive extracytoplasmic function-sigma (ECF-σ) factor SigH.

## Introduction

Microbes have evolved a number of strategies to face the challenges of changing environments and survive under conditions of stress. One of these strategies is to synthesize low-molecular-weight (LMW) thiols that act as the most important cellular antioxidants and detoxifiers to protect cells from toxic xenobiotics [1]. Eukaryotes and Gram-negative bacteria produce the tripeptide glutathione (GSH; γ-L-glutamyl-L-cysteinylglycine) as the LMW thiol redox buffer, whereas some Gram-positive bacteria, such as members of *Corynebacterium*, *Mycobacterium*, *Rhodococcus* and *Streptomyces*, cannot produce GSH but instead synthesize its functional equivalent, mycothiol (MSH; AcCys-GlcN-Ins) [2], [3]. Like GSH, MSH plays a key role in protecting the cell against environmental stresses such as antibiotics, alkylating agents, oxidants, heavy metals and extreme pH [4]–[6]. Therefore, MSH is considered as a general protection agent for improving the robustness and the survival of cells facing environmental stress conditions.

Mycothiol *S*-conjugate amidase (Mca) is a key enzyme involved in MSH-dependent detoxification, which was first isolated from the acid-fast bacterium *Mycobacterium smegmatis* [7]. In detoxification process, mycothiol directly reacts with electrophilic compounds by its thiol moiety, forming MSH *S*-conjugates (MSRs) [8]. MSR is then cleaved by Mca to release *N*-acetylcysteine (AcCys) *S*-conjugate (a mercapturic acid) into the medium and retain glucosaminyl inositol (GlcN-Ins) in the cell to regenerate MSH [8]. Mca has been reported to have extensive substrate specificity towards alkylating agents including *S*-conjugates of monobromobimane (mBBr), iodoacetamide (IAM) and *N*-ethylmalemide (NEM), and antibiotics such as cerulenin and rifampin [8]. Any compound, whether electrophilic or non-polar, that contains aromatic rings, α, β-unsaturated ketones, epoxide rings, arene oxides, or quinines structure, is considered capable to conjugate with MSH [9], [10]. These chemical structures also exist in some antibiotics. Interestingly, some antibiotic biosynthetic operons, such as erythromycin and lincomycin, also contain Mca homologs [11]. Diverse mercapturic acids of antibiotics have been detected in the broth of some antibiotic-producing actinomycetes [12]. These findings have led to the proposal that these antibiotics and electrophilic compounds can be conjugated to MSH and are detoxified by Mca. Recently, plenty of marine natural products inhibiting Mca have been identified; some of them are lethal to *M. smegmatis* and play potential roles in chemical synthesis of drugs directed against Mca [13]–[15].

Recently, we have reported that *C. glutamicum* mutants lacking MSH are more sensitive to some oxidative agents, electrophiles, and antibiotics, but MSH cannot directly remove these substrates [16]. A search of GenBank sequence database revealed that *C. glutamicum* Mca, encoded by an annotated *mca* gene, has higher amino acid sequence identities with Mca of *M. smegmatis* and *M. tuberculosis* (about 30%). To better understand the role of Mca in xenobiotic detoxification, model organism *C. glutamicum* was used to provide a certain basis for the study of pathogenic microorganisms. In this study, the potential roles of Mca in the survival of *C. glutamicum* by coping with multiple stresses and detoxifying toxins were investigated. Besides, the enzyme was characterized in terms of its metal ion requirement, substrate specificity and regulation mechanism, thus contributing to a deeper understanding of the important enzymatic detoxifier and drug target.

## Materials and Methods

### Bacterial strains and culture conditions

Bacterial strains used in this study are listed in S1 Table. *Escherichia coli* and *C. glutamicum* strains were routinely cultured in Luria-Bertani (LB) broth or on LB plates at 37°C and 30°C, respectively. For generation of mutants and maintenance of *C. glutamicum*, brain heart infusion broth with 0.5 M sorbitol (BHIS) was used. The *C. glutamicum* RES167 strain was the parent of all derivatives used in this study. In-frame deletions were generated by the method as described previously [17]. Cell growth was monitored by measuring absorbance at 600 nm (*A*_600_). Antibiotics were added at the following concentrations: kanamycin, 50 µg ml^−1^ for *E. coli* and 25 µg ml^−1^ for *C. glutamicum*; nalidixic acid, 40 µg ml^−1^ for *C. glutamicum*; chloramphenicol, 20 µg ml^−1^ for *E. coli* and 10 µg ml^−1^ for *C. glutamicum*.

### Plasmid construction

The genes encoding *C. glutamicum* Mca (NCgl0948) and RNA polymerase sigma factor RpoE (SigH, NCgl0733) were amplified by PCR using *C. glutamicum* RES167 genomic DNA as template with primers listed in S1 Table. The resulting DNA fragments were digested and afterwards subcloned into similar digested pET28a vectors, obtaining plasmids pET28a-*mca* and pET28a-*sigH*, respectively. The plasmid pK18*mobsacB-Δmca* was used to construct the *C. glutamicum mca* deletion mutant. The 786 bp upstream PCR product and 810 bp downstream PCR product of *mca* were amplified using primer pairs DMcaF1/DMcaR1 and DMcaF2/DMca-R2, respectively. In the next step, the upstream and downstream products were digested with BamHI and SalI, respectively, and inserted into BamHI/SalI sites of pK18*mobsacB* to get pK18*mobsacB-Δmca*. Plasmids pK18*mobsacB*-*ΔsigH* was constructed in a similar approach using primers listed in S1 Table. To complement the *mca* mutant, primers CMcaF/CMcaR were used to amplify the *mca* gene fragment from *C. glutamicum* RES167 genome. The PCR product of *mca* was digested with BamHI/EcoRI and inserted into the BamHI/EcoRI sites of pXMJ19 to produce pXMJ19-*mca*. The pXMJ19-*mca* plasmid was transformed into relevant *C. glutamicum* strains by electroporation. Expression in *C. glutamicum* was induced by addition of 0.5 mM isopropyl β-D-1-thiogalactopyranoside (IPTG). The *lacZ* fusion reporter plasmid pK18*mobsacB-P_mca_::lacZ* was constructed by fusion of the *mca* promoter to the *lacZY* reporter gene via overlap PCR [18]. In the first round of PCR, a 1000 bp *mca* promoter DNA fragment and the *lacZY* DNA fragment were amplified with the primer pairs pMca-F1/pMca-R and lacZY-F/lacZY-R, respectively. These fragments were used as template in the second round of PCR with primers pMca-F and lacZY-R. The resulting PCR fragments were digested with SmaI/PstI and inserted into the suicide vector pK18*mobsacB* to get pK18*mobsacB-P_mca_::lacZ* fusion construct [18]. Site-directed mutagenesis of Mca was carried out by overlap PCR as described [19]. To replace Asp residue at position 14 with Ala residue (D14A), the mutant *mca:D14A* DNA segment was amplified by two rounds of PCR. Primer pairs DMcaF1/McaD14AR and McaD14AF/DMcaR2 were used to amplify segments 1 and 2, respectively. The second round of PCR was carried out using EMcaF/EMcaR as primer pair and segments 1 and 2 as templates to get *mca:D14A* segment, which contained mutation at the ^14^D site of Mca. The *mca:D14A* DNA fragment was digested and cloned into similar digested pET28a to generate pET28a-*mca:D14A* plasmid. Similarly, *mca:H10A, mca:H12A*, *mca:D15A*, *mca:E16A*, *mca:E43A*, *mca:D132A*, *mca:Y137A*, *mca:H139A*, *mca:D141A* and *mca:H142A* segments were obtained with primers listed in S1 Table. These DNA fragments were cloned into pET28a to generate corresponding plasmids for expression. The fidelity of all the constructs was confirmed by DNA sequencing (Sangon Biotech, Shanghai, China).

### Heterologous expression and purification of recombinant proteins

To express and purify His_6_-tagged proteins, recombinant pET28a plasmids were transformed into the *E. coli* BL21(DE3) host. Recombinant strains were grown at 37°C in LB broth to *A*_600_ of 0.4, shifted to 22°C, induced with 0.4 mM isopropyl β-D-1-thiogalactopyranoside (IPTG) and grown for additional 12 h. Harvested cells were disrupted by sonication and then purified with Ni-NTA His⋅Bind Resin (Novagen, Madison, WI) based on manufacturer’s instructions. Protein samples were run on 15% SDS-PAGE and visualized by Coomassie brilliant blue staining. Purified recombinant proteins were dialyzed in phosphate-buffered saline (PBS) overnight at 4°C and then stored at −80°C until used. Protein concentrations were determined using the Bradford Protein Assay Kit (Bio-Rad, Hercules, CA) on the manufacturer’s instructions, with bovine serum albumin (BSA) as standard.

### Molecular weight determination

The native molecular weight of recombinant His_6_-Mca was determined by gel filtration using a high performance liquid chromatography (HPLC) system equipped with a HiLoad 26/600 Superdex 200 GL column (GE Healthcare, Piscataway, NJ). The column was pre-equilibrated with 50 mM potassium phosphate buffer (pH 7.2) containing 0.15 M NaCl. The elution volumes were used to calculate the *K*_av_ values for each of the standard protein *K*_av_ = (*V*_e_–*V*_0_)/(*V*_t_–*V*_0_), where *V*_0_ is the void volume of the column, *V*_t_ is the total volume of the column, and *V*_e_ is the elution volume of the protein). The resulting *K*_av_ was drawn against the molecular mass of the standard proteins to plot the standard curve. The data were fitted with a linear equation. The following protein standards were used (GE Healthcare): aprotinin (6.5 kDa), ribonuclease A (13.7 kDa), carbonic anhydrase (29 kDa), ovalbumin (44 kDa), conalbumin (75 kDa), and aldolase (158 kDa).

### Deacetylase and amidase activity assays

Deacetylase activity of *C. glutamicum* Mca was analyzed by assessing the formation of glucosamine (GlcN) using the method of Huang and Hernick [20] with minor modifications. The reaction mixture (350 µl) containing 50 mM HEPES, 50 mM NaCl, 1.0 mM tris(2-carboxyethyl)phosphine (TCEP, pH 7.5) and 2.0 mM *N*-acetyl-D-glucosamine (GlcNAc) was pre-incubated at 30°C before the addition of Mca protein (1.0 µM) to initiate the reaction. After various time intervals, reaction aliquots (60 µl) were terminated by the addition of trichloroacetic acid (5%). After centrifugation (13,000 rpm, 5 min), the supernatant (30 µl) was collected, diluted with borate solution (0.75 M, pH 9.0) and labeled with fluorescamine (FSA, 2.3 mM). After 30 min (fluorescence signal remained stable for up to 1 h), the resulted fluorescence was measured (excitation 395 nm, emission 485 nm) using a SpectraMax M5 plate reader (Molecular Devices, Sunnyvale, CA). The observed increase in fluorescence ([FU]/min) was converted into µM min^−1^ according to the GlcN standard curve. To determine steady-state parameters, enzyme activity was measured at eight different concentrations of GlcNAc (0–5.0 mM), and kinetic parameters *k*_cat_, *K*_m_, and *k*_cat_/*K*_m_ were obtained by fitting the Michaelis-Menten equation to the initial linear velocities using the curve-fitting program Kaleidagraph (Synergy Software), which also calculates the asymptotic standard errors.

Similarly, amidase activity of *C. glutamicum* Mca was analyzed by assessing the formation of GlcN at 37°C using mycothiol *S*-conjugate of mBBr (MSmB) as substrate [20]. The reaction mixture (100 µl) included 50 mM HEPES (pH 7.5), 50 mM NaCl, 0.1 mM dithiothreitol (DTT) and 1.0 mM MSmB, and 2.0 µM Mca. After 60 min, the reaction was terminated by addition of an equal volume of acetonitrile followed by incubation at 60°C for 10 min. After centrifugation (13,000 rpm, 5 min), the supernatant (30 µl) containing GlcN was analyzed as described above. MSmB was prepared by derivatization with mBBr through the method described previously [7].

### Effects of pH on Mca activity

pH-dependence experiments were conducted on *C. glutamicum* Mca with 50 mM buffer solutions (MES, pH 6–6.8; MOPS, pH 6.5–7.5; HEPES, pH 7.3–8.8; *N*-bis(2-hydroxyethyl)-glycine, pH 8–9; borate, pH 9–10; carbonate, pH 10–11) containing 1 mM TCEP. The method was the same as deacetylase and amidase activity assays. Steady-state kinetic parameters *K*_m_, *k*_cat_, and *k*_cat_/*K*_m_ for protein activity were determined by fitting initial velocities to the Michaelis-Menten equation. The following equation was fitted to the pH rate profile.
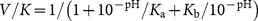
(1)where *V* is the observed rate of the reaction, *K* is pH-dependent rate constant for GlcNAc and MSmB substrates, and *K*_a_ and *K*_b_ are the ionization constants of the acid and base species, respectively [21].

### Preparation of metal-free Mca and metal reconstitution

Metal-free Mca and reconstituted Mca with the desired metal ions were prepared as previously described [22]. Briefly, purified protein (100 µM) was added to the solution containing 25 mM Tris, 25 mM diethylene triamine pentaacetic acid, and 10% glycerol, at pH 7.5 and put on ice. After 1 h, the protein solution was dialyzed with buffer (25 mM Tris, 10% glycerol, pH 7.5) at 4°C. The concentration of residual metal ions was determined by atomic absorption spectrometry (ZEEnit 650P, Analytik Jena, Germany). For reconstitution with metal ion, apo-Mca (10 µM) was added to a stoichiometric concentration of the desired divalent-metal ions (Co^2+^, Fe^2+^, Mn^2+^, Ni^2+^ and Zn^2+^) and put on ice for 30 min. To determine the optimal metal/protein ratio, apo-Mca was incubated with various concentrations of the metal ions (0–25 µM) for 30 min on ice prior to activity assays. These solutions were dialyzed again as mentioned above to remove unbound metal ions and then analyzed by atomic absorption spectrophotometry.

### Sensitivity assays for antibiotics, heavy metals, alkylating agents and oxidants

Disk diffusion assays were performed for antibiotics, alkylating agents, and oxidative agents according to Rawat et al. [5]. Briefly, cells were grown to the mid-log phase and a lawn of cells was plated onto LB plates. After the paper disks were placed into the plates, various amounts of agents (10 µl) were added to the disk. The disks were allowed to dry and the plates were incubated for 2 to 3 days. For the minimal broth dilution assay, antibiotics were serially diluted (0.5×) in LB medium (1 ml) and cells (*A*_600_ = 1.6) were inoculated. After 1 to 2 days of incubation at 30°C, the tubes were checked for growth. The bacteriostasis growth curve assay was used to determine the sensitivity of *C. glutamicum* to heavy metal stress. LB-grown strains (50 µl) were transferred to LB broth (5 ml), with and without addition of various concentrations of heavy metals (Cd^2+^, Ni^2+^, Cr^2+^ and Cu^2+^) and incubated at 30°C. The cellular growth was monitored turbidimetrically (*A*_600_) after 24 h. All assays were performed in triplicate.

### MSH purification

MSH was purified from *C. glutamicum* RES167 with thiopropyl sepharose 6B column followed by Sephadex LH-20 (GE Healthcare, Piscataway, NJ) chromatography as described by Feng et al. [23]. The concentration of purified MSH was measured by thiol-specific fluorescent-labeling HPLC method [24] with GSH as thiol standard. The HPLC system was equipped with an Extend-C18 column (250×4.6 mm, Beckman Ultrasphere ODS IP column; Beckman Coulter, Brea, CA) operated with acetic acid-methanol gradient elution at the flow rate of 0.9 ml min^−1^. The bimane derivative of MSH was eluted at approximately 15 min in this system.

### Analysis of amidase activity with mBBr as substrate *in vivo*

The *in vivo* amidase activity of Mca with mBBr as substrate was performed as previously described [8]. Briefly, triplicate samples (10 ml each) of exponentially growing *C. glutamicum* strains were chilled on ice for 20 min followed by addition of mBBr (0.5 mM in acetonitrile) and further incubated on ice for 30 min. Excess amount of 2-mercaptoethanol (1 mM) was added to scavenge unreacted mBBr. Cells were harvested and extracted with 50% acetonitrile at 60°C for 10 min. After acidifying with methanesulphonic acid, cell debris was removed by centrifugation and the supernatant was analyzed by HPLC. To determine the amount of acetylcysteinyl bimane (AcCysmB) in the medium, the culture supernatant was analyzed by HPLC system equipped with a Beckman Ultrasphere ODS IP column (4.6×25 cm; Beckman Coulter, Brea, CA). MSmB and AcCysmB were eluted with gradients of eluent A (0.25% acetic acid, pH 3.6 with NaOH) and eluent B (methanol). The proportion of buffer in continuous gradients was as follows: 10%, 0–5 min; 10–50%, 5–15 min; and 50–100%, 15–30 min. The MSmB standard (2 mM) was prepared as described [7]. The concentration of bimane derivatives was presented as µmol g^−1^ dry cell weight.

### Chromosomal fusion reporter construction and β-Galactosidase assay

The *lacZ* fusion reporter plasmid pK18*mobsacB-P_mca_::lacZ* was transformed into the WT(pXMJ19), *ΔsigH*(pXMJ19), and *ΔsigH*(pXMJ19-*sigH*) strains by electroporation, and the chromosomal pK18*mobsacB-P_mca_::lacZ* fusion reporter strains were selected by plating on LB-kanamycin plates. The resulted strains were grown in LB medium to an optical density at 600 nm (*A*_600_) of 0.6–0.7 and then treated with different toxic agents of various concentrations at 30°C for 30 min. β-Galactosidase activity was assayed with *o*-nitrophenyl-β-galactoside as substrate [25]. Data were presented as the means of triplicate experiments with error bars representing the standard deviation.

### RNA isolation and quantitative real-time PCR (qRT-PCR) analysis

Total RNA was isolated from exponentially growing WT(pXMJ19), *ΔsigH*(pXMJ19) and *ΔsigH*(pXMJ19-*sigH*) strains exposed to different toxic agents of indicated concentrations for 30 min using the RNeasy Mini Kit (Qiagen, Hilden, Germany) along with the DNase I Kit (Sigma-Aldrich, Taufkirchen, Germany). Purified RNA was reverse-transcribed with random 9-mer primers and MLV reverse transcriptase (TaKaRa, Dalian, China). Quantitative RT-PCR analysis was performed using 7500 Fast Real-Time PCR System (Applied Biosystems, Foster City, CA) as described previously [26]. The primers used are listed in S1 Table. The relative abundance of the target mRNAs was quantified based on the cycle threshold value. To standardize the results, the relative abundance of 16 S rRNA was used as an internal standard.

### Electrophoretic mobility shift assay (EMSA)

EMSA was performed through the method described by Zhang et al. [19] with modifications. Briefly, DNA probes (400 bp *P_mca_* fragments) were amplified from the *mca* promoter region of corresponding pk18*mobsacB*-*P_mca_::lacZ* reporter vectors using primers *Pmca*-F2 and *Pmca*-R (S1 Table). The reaction mixture (10 µl) contained 20 mM Tris-HCl (pH 7.4), 4 mM MgCl_2_, 100 mM NaCl, 1 mM dithiothreitol, 10% (v/v) glycerol, 20 ng DNA probes and 0–3.0 µg of purified His_6_-SigH. After incubation for 30 min at room temperature, the binding reaction mixture was subjected to electrophoresis on 6% native polyacrylamide gel containing 5% glycerol in 0.5×TBE buffer, and the DNA probe was detected with SYBR Green (Promega, Fitchburg, WI).

## Results

### Roles of *C. glutamicum mca* in resistance to alkylating agents and oxidants

It was shown previously that MSH reacts with alkylating agents, such as 1-chloro-2,4-dinitrobenzene (CDNB), mBBr, IAM, NEM and methylglyoxal (MG), to form conjugates as that for *Streptomyces coelicolor* Mca [8]. Moreover, MSH takes part in the detoxification of alkylating agents in *C. glutamicum*, as mutants lacking MSH are more susceptible to these agents than the wild type [16]. Thus, the sensitivity of RES167 wild type and the *Δmca* mutant to alkylating agents was examined. As shown in Table 1, *Δmca* was more susceptible to mBBr, IAM, NEM, CDNB and MG than the wild type strain, showing 0.60-, 0.92-, 0.59-,1.89- and 1.1-fold increase in the size of growth inhibition zones, respectively, suggesting that MSH-mBBr, MSH-IAM, MSH-NEM, MSH-CDNB and MSH-MG adducts were formed that are substrates of Mca. This assumption was further confirmed by the observation that the sensitivity phenotypes of the *mca* mutant was fully recovered in the complementary strain *Δmca*(pXMJ19-*mca*) (Table 1).

**Table 1:** Sensitivity of *C*. *glutamicum* strains to alkylating agents tested by disk diffusion assay.

Alkylating agents	Concentration (mM)	Size of growth inhibition zone (cm) of various strains^a^		
		WT(pXMJ19)	*Δmca*(pXMJ19)	*Δmca*(pXMJ19-*mca*)
mBBr	90	1.5±0.3	2.4±0.4*	1.6±0.4
IAM	0.54	1.2±0.5	2.3±0.5*	1.2±0.3
NEM	100	1.7±0.3	2.7±0.4*	1.9±0.5
CDNB	49.4	0.9±0.1	2.6±0.1**	resistant
MG	20	2.1±0.3	4.4±0.4*	2.3±0.2

mBBr, monobromobimane; IAM, iodoacetamide; NEM, *N*-ethylmaleimide; CDNB, 1-chloro-2,4-dinitrobenzene MG, methylglyoxal. *P≤0.05 or **P≤0.01 versus wild type for the mutants.

a The values are mean±SD for three independent determinations.

Rawat et al. [11] demonstrated that MSH-deficient *M. smegmatis* mutants are more sensitive to redox cycling agents, such as menadione (MD), plumbagin and nitrofurantoin. Similarly, *C. glutamicum* mutants lacking MSH were more sensitive to some oxidants, including H_2_O_2_, formaldehyde and diamide [16]. To investigate whether *C. glutamicum* detoxifys oxidants via MSH-dependent Mca pathway, the sensitivity of *Δmca* to various oxidative agents was determined by disk diffusion assays (Table 2). Results showed that the *Δmca* was significantly more sensitive than the wild type strain to MD (*p*<0.01), but there was no difference in its sensitivity to the low concentration of hydrogen peroxide (H_2_O_2_), cumene hydrogen peroxide (CHP), formaldehyde and diamide (*p*>0.05). However, a slight difference in the sensitivity between the wild type and *Δmca* was observed at high concentrations of H_2_O_2_, CHP and diamide (Table 2). Importantly, all these sensitivity phenotypes were restored in the complementary strain *Δmca*(pXMJ19-*mca*). In contrast, there was no difference in sensitivity to either high or low concentration of DTT between the wild type and *Δmca* (Table 2). These results consolidate the previous findings in *M. smegmatis* [11].

**Table 2:** Sensitivity of *C*. *glutamicum* strains to oxidizing and reducing agents tested by disk diffusion assay.

Agents	Concentration (mM)	Size of growth inhibition zone (cm) of various strains^a^		
		WT(pXMJ19)	*Δmca*(pXMJ19)	*Δmca*(pXMJ19-*mca*)
Hydrogen peroxide	1.00	1.6±0.3	1.8±0.4	1.6±0.4
	9.98	2.9±0.4	3.4±0.4	2.7±0.3
Formaldehyde	1.00	3.4±0.3	3.5±0.5	3.5±0.2
	3.98	5.1±0.3	5.3±0.4	5.3±0.3
Diamide	1.00	1.6±0.3	1.7±0.3	1.6±0.4
	5.00	2.3±0.3	2.7±0.3	2.5±0.4
Cumene hydrogen peroxide	1.00	2.5±0.4	2.8±0.3	2.1±0.4
	5.50	3.7±0.2	4.1±0.1	3.9±0.4
Menadione	50	1.0±0.2	2.2±0.3**	1.1±0.2
Dithiothreitol	0.30	1.1±0.4	1.0±0.3	1.2±0.4
	1.00	2.1±0.3	2.0±0.2	2.2±0.1

**P≤0.01 versus wild type for the mutant.

a The values are mean±SD for three independent determinations.

### Role of *C. glutamicum mca* in resistance to antibiotics

It was reported that *M. smegmatis* Mca is involved in antibiotics resistance by cleaving MSH *S*-conjugates of rifamycin S [27] and cerulenin [28], resulting in the excretion of mercapturic acid [27], [28]. To test whether *C. glutamicum* Mca is also involved in detoxification of antibiotics, sensitivity test was performed on the RES167 wild type, *Δmca* and the complementary strain *Δmca*(pXMJ19-*mca*) against six types of antibiotics with different chemical structures.

First, sensitivity of *C. glutamicum* strains to Macrolides and β-Lactams was tested. As shown in Table 3, *Δmca* was more sensitive to rifamycin S than the wild type, as indicated by the larger size of growth inhibition zone (*p*<0.01). This result was further confirmed with the more sensitive minimal broth dilution assay [27], in which the minimum inhibitory concentration (MIC) of rifamycin S for WT(pXMJ19) was 4-fold higher than that for *Δmca*(pXMJ19) (Table 4). These observations indicate that the Mca-dependent detoxification pathway indeed plays a major role in the detoxification of rifamycin S. However, no significant difference was found in the size of growth inhibition zones around erythromycin, lincomycin and spectinomycin (all belong to macrolides as rifamycin S) between WT(pXMJ19), *Δmca*(pXMJ19) and the complementary strain *Δmca*(pXMJ19-*mca*) (Table 3; *p*>0.05). Similar trend was obtained in the MIC data (Table 4). Similarly, for penicillin of β-Lactams, no significant difference among WT(pXMJ19), *Δmca*(pXMJ19) and *Δmca*(pXMJ19-*mca*) was observed in either the size of growth inhibition zones or MIC values (Tables 3&4).

**Table 3:** Sensitivity of *C. glutamicum* strains to various classes of antibiotics tested by disk diffusion assay.

Antibiotics	Concentration (mg ml^−1^)	Size of growth inhibition zone (cm) of various strains^a^		
		WT(pXMJ19)	*Δmca*(pXMJ19)	*Δmca*(pXMJ19-*mca*)
**Macrolide**				
Erythromycin	0.5	1.9±0.4	2.0±0.5	2.1±0.4
Lincomycin	25	3.2±0.4	3.4±0.2	3.4±0.2
Rifamycin S	0.25	1.3±0.3	2.5±0.2**	1.5±0.3
Spectinomycin	3	0.9±0.2	1.0±0.3	1.0±0.5
**β-Lactam**				
Penicillin	90	2.1±0.3	2.2±0.4	2.1±0.2
**Glycopeptide**				
Vancomycin	1.25	0.9±0.5	1.8±0.4**	1.1±0.3
**Aminoglycoside**				
Streptomycin	50	2.3±0.3	2.8±0.4	2.6±0.4
Neomycin	60	1.5±0.3	1.9±0.2	1.6±0.3
Gentamycin	10	1.9±0.4	2.2±0.6	2.0±0.3
**Quinolone**				
Ciprofloxacin	5	1.3±0.2	3.2±0.3**	2.1±0.2
**Tetracycline**				
Tetracycline	30	1.3±0.3	3.1±0.2**	2.3±0.2

**P≤0.01 versus wild type for the mutants.

a The values are mean±SD for three independent determinations.

**Table 4:** The minimum inhibitory concentrations (MICs) of various antibiotics for *C*. *glutamicum* strains.

Antibiotics	MIC (µg ml^−1^)		
	WT(pXMJ19)	*Δmca*(pXMJ19)	*Δmca*(pXMJ19-*mca*)
**Macrolide**			
Erythromycin	25.00	25.00	25.00
Lincomycin	62.50	62.50	62.50
Rifamycin S	12.50	3.125	6.25–3.125
Spectinomycin	128.00	128.00	128.00
**β-Lactam**			
Penicillin	>350.00	>350.00	>350.00
**Glycopeptide**			
Vancomycin	32.00	16.00–8.00	32.00–16.00
**Aminoglycoside**			
Streptomycin	1.25	0.3125–0.156	0.625
Neomycin	7.50	3.75	7.50
Gentamycin	5.00	5.00	5.00
**Quinolone**			
Ciprofloxacin	1.562	0.391	1.562–0.781
**Tetracycline**			
Tetracycline	3.125	0.781	1.562

**P≤0.01 versus wild type for the mutants.

a The values are mean±SD for three independent determinations.

Recent studies have reported that MSH-deficient mutants are more sensitive to vancomycin of glycopeptides [29], [30]. To determine whether this sensitivity depends on Mca, the sensitivity of *C. glutamicum* strains to vancomycin was tested. A clear difference was detected in the size of growth inhibition zone around vancomycin disk (Table 3), with about 2–4 fold difference in the corresponding MIC values between *Δmca*(pXMJ19) and WT(pXMJ19)/*Δmca*(pXMJ19-*mca*) (Table 4).

Interestingly, *Δmca*(pXMJ19) was also more sensitive to ciprofloxacin (quinolone) and tetracycline than WT(pXMJ19) and *Δmca*(pXMJ19-*mca*). The observed MICs of WT(pXMJ19) and *Δmca*(pXMJ19-*mca*) strains to ciprofloxacin and tetracycline were about 4-fold higher than that of *Δmca*(pXMJ19) (Table 4). These results were consistent with the differences in the size of growth inhibition zones around ciprofloxacin (1.5-fold) and tetracycline disks (1.4-fold) between WT(pXMJ19)/*Δmca*(pXMJ19-*mca*) and *Δmca*(pXMJ19) (Table 3).

As for aminoglycosides, there was insignificant difference in the size of growth inhibition zones around streptomycin and neomycin disks among WT(pXMJ19), *Δmca*(pXMJ19) and *Δmca*(pXMJ19-*mca*) strains (Table 3). However, in the more sensitive minimal broth dilution assay [27], both WT(pXMJ19) and *Δmca*(pXMJ19-*mca*) showed significant higher MIC values than *Δmca*(pXMJ19) for streptomycin and neomycin but not gentamycin (Table 4). Overall, the results of sensitivity assays supported the hypothesis that Mca plays a role in detoxifying a number of antibiotics in *C. glutamicum*.

### Role of *C. glutamicum mca* in resistance to heavy metals

**Figure 1:**
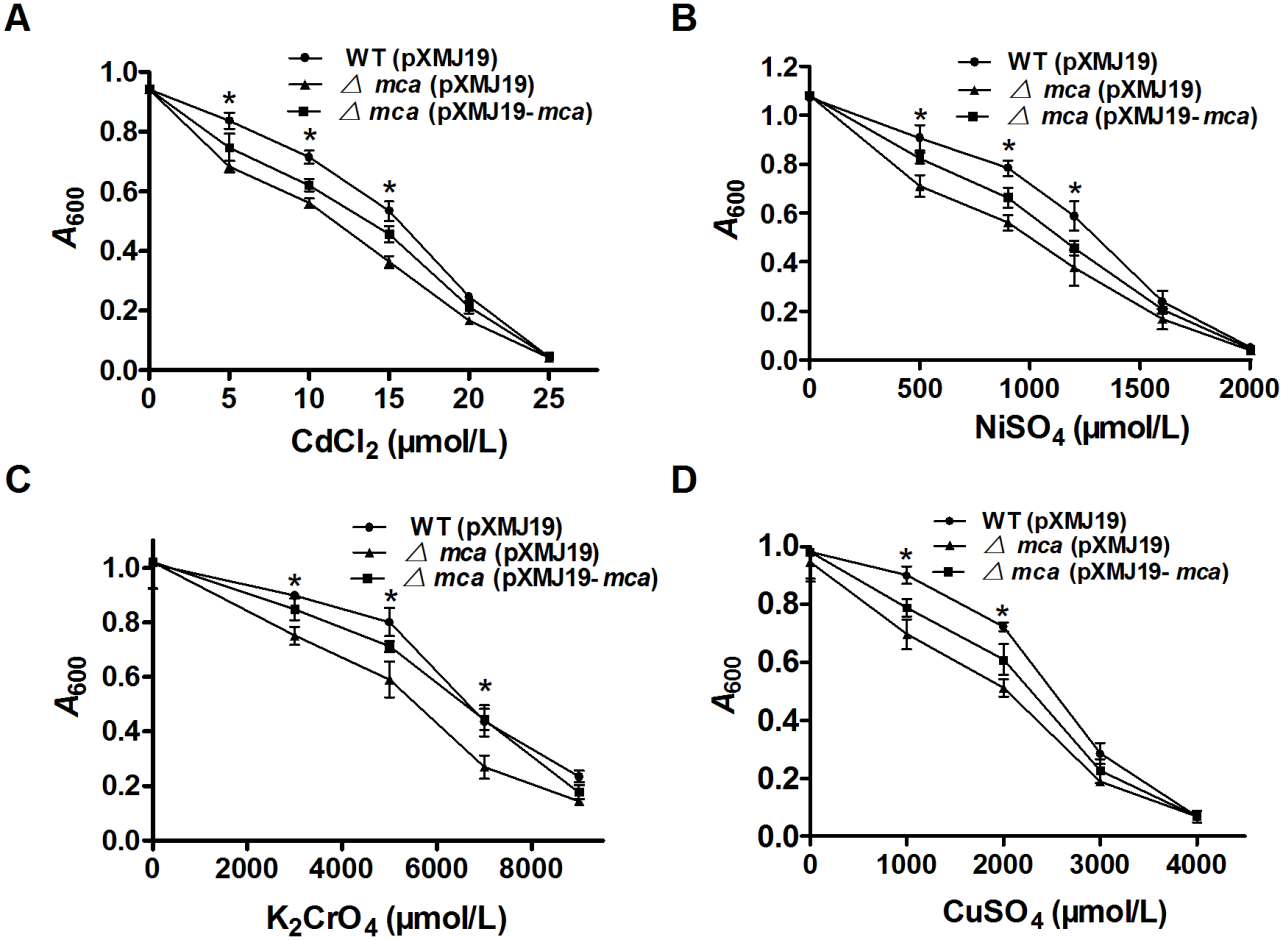
Sensitivity assays of *C. glutamicum* strains to heavy metals. **A–D** The growth (*A*_600_) of the *C. glutamicum* WT(pXMJ19), *Δmca*(pXMJ19) and *Δmca*(pXMJ19-*mca*) strains after 24 h at 30°C in LB medium containing increasing concentrations of Cd^2+^(A), Ni^2+^ (B), Cr^2+^(C) and Cu^2+^(D) was recorded. Mean values with standard deviations (error bars) from at least three repeats are shown. *: *P≤*0.05.

Heavy metal ions, including Cd^2+^, Ni^2+^, Cr^2+^ and Cu^2+^, markedly inhibited growth of the *mca* mutant relative to the wild type strain, and the growth inhibition phenotype was complemented in the *Δmca*(pXMJ19-*mca*) strain by plasmid-borne *mca* expression (Fig. 1). These results suggested that Mca is also involved in heavy metal resistance in *C. glutamicum*.

### Detoxification of mBBr by Mca *in vivo*

Since mBBr easily permeates into cells and subsequently converts intracellular MSH to its bimane derivatives (MSmB) [31], mBBr was chosen as substrate to examine whether *C. glutamicum* Mca could indeed achieve detoxification by cleaving the amide bond between MSH and toxins *in vivo*.

**Figure 2:**
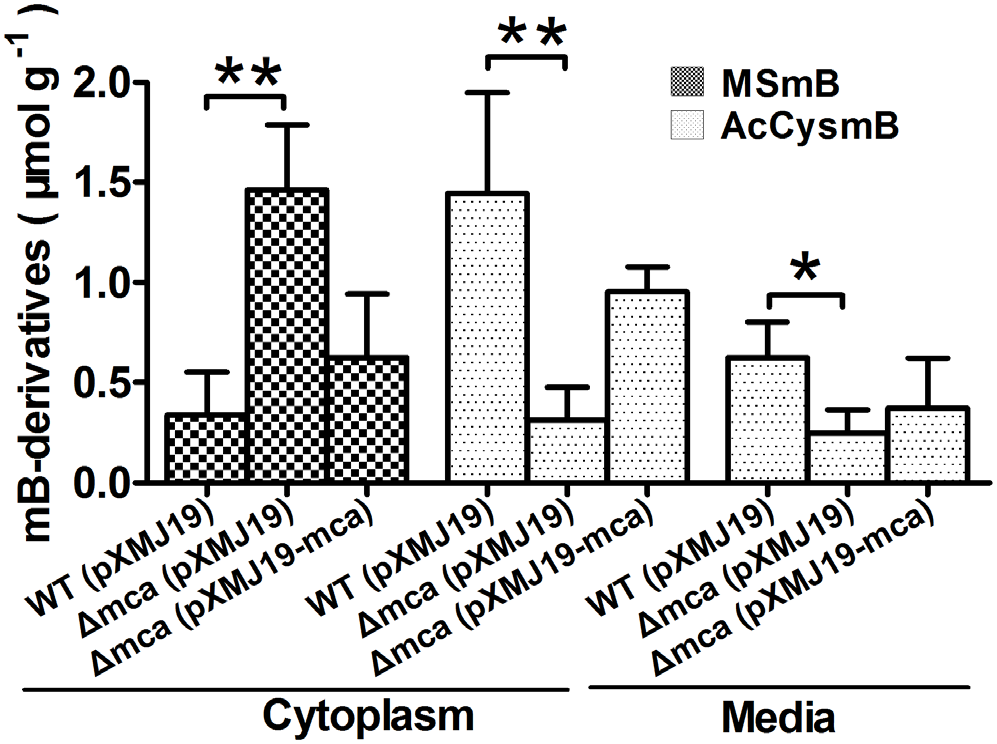
The amidase activity of *C. glutamicum* Mca. The levels of reactant (MSmB) and product (AcCysmB) of the amidase activity following reaction of mBBr and MSH in different *C. glutamicum* strains were determined. Cells grown in LB (*A*_600_ = 1.6) were treated with monobromobimane (mBBr), to form a fluorescent bimane derivative of mycothiol (MSmB). Mca cleaved MSmB to AcCysmB (*N*-acetylcystein *S*-conjugate of bimane) that was released into the medium. The amounts of MSmB and AcCysmB in the cytoplasm and the medium were measured by HPLC and were presented as µmol g^−1^ dried cell weight. Mean values with standard deviations (error bars) from at least three repeats were shown. **: *P≤*0.01. *: *P≤*0.05.

As expected, the MSmB substrate was almost completely transformed into AcCysmB, the product of the Mca reaction, in the wild type cells. On the contrary, the amount of MSmB remained unchanged in the *Δmca* cells. Introduction of the *mca* gene to the mutant through pXMJ19 vector partially restored its ability to transform MSmB into CysmB (Fig. 2), further confirming the amidase activity of Mca. Consistently, significant higher level of AcCysmB leaked into medium was observed for the wild type WT(pXMJ19) and the complementary strain *Δmca*(pXMJ19-*mca*) compared to *Δmca* (Fig. 2). These results indicate that Mca improves *C. glutamicum* resistance to various toxins by catalyzing the hydrolysis of the cysteinyl-glucosamine amide bond in mycothiol *S*-conjugates, as similar as the *S. coelicolor* Mca [8].

### Monomeric structure of Mca

The open reading frame of the putative *C. glutamicum mca* gene (*ncgl0948*) was cloned into pET28a vector and expressed in *E. coli* BL21(DE3). The resulted strain induced by IPTG showed protein over-expression at around 35.0 kDa in SDS-PAGE. After cell sonication and protein separation into fractions by centrifugation, Mca was retained in the soluble fraction. The purified recombinant Mca showed a single band in SDS-PAGE gel (S1A Figure). The oligomerization properties of purified Mca were examined by analytical gel-filtration chromatography (S1B&C Figures). In the gel-filtration chromatogram, a sharp peak appeared at the elution time of 5.5 min (S1C Figure). According to the standard curve (S1B Figure), the native molecular mass of Mca was estimated to be 35 kDa, closer to the value deduced from its amino acid sequence (33 KDa). This result indicates that Mca eluted from the column is entirely monomeric. Because a protein could switch between monomer and multimer when binding to different ligands [32], [33], so it doesn’t completely exclude the possibility that Mca may also exist as oligomers *in vivo* upon ligand binding.

### Deacetylase and amidase activitites of Mca

**Figure 3:**
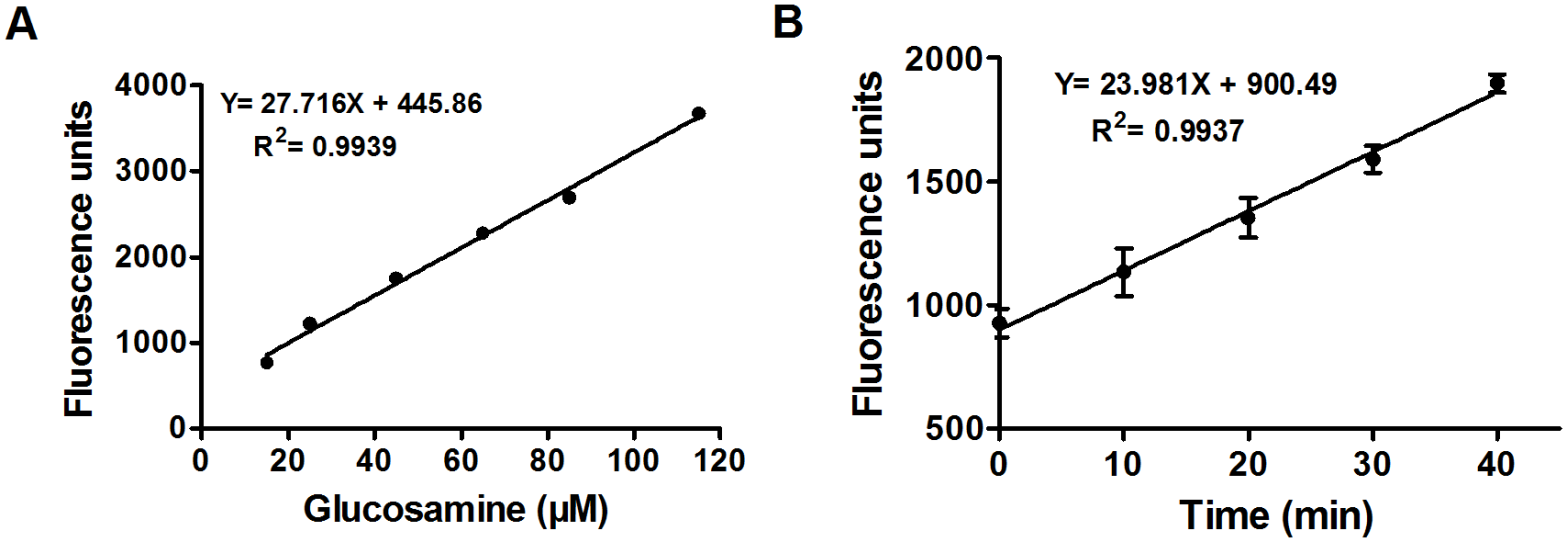
Catalytic activity of *C. glutamicum* Mca. **A.** GlcN standard curve. Solutions of GlcN (0–100 µM) in buffer (50 mM HEPES, 50 mM NaCl, and 1 mM TCEP, pH 7.5) were diluted with borate (0.75 M, pH 9.0) and mixed with FSA (2.3 mM), followed by measurement of the resulting fluorescence. The observed increase in fluorescence was drawn against glucosamine concentration to generate the standard curve. A linear equation was fitted to data. **B.** Mca-catalyzed reaction. GlcNAc (2 mM) was pre-incubated at 30°C in assay buffer (50 mM HEPES, 50 mM NaCl, and 1 mM TCEP, pH 7.5) and the reaction was started by the addition of Mca (6.4 µM). At different time points, aliquots of reaction mixture were terminated by 5% trichloroacetic acid, diluted with borate (0.75 M, pH 9.0) and mixed with FSA (2.3 mM), followed by measurement of the resulting fluorescence. The glucosamine standard curve (**A**) was used to transform the observed rate of the reaction into µ min^−1^.

A close homologue of Mca is the GlcNAc-Ins deacetylase of *M. tuberculosis* (MshB) that cleaves amide bond of GlcNAc-Ins and shares similar substrate specificities with Mca [21]. A recent report demonstrated that *mshB* null mutant of *C. glutamicum* can still accumulate a certain amount of MSH [16], indicating that some other enzymes possibly have deacetylase activity to catalyze GlcNAc-Ins in *C. glutamicum*. Thus, *C. glutamicum* Mca, having higher identity with MshB, may be a reasonable candidate possessing deacetylase activity (S2 Figure). To test this possibility, FSA-based assay was used to measure the steady-state turnover of GlcNAc by Mca. As reported, FSA reacted with amines of GlcN to form a fluorescent product (excitation 395 nm, emission 485 nm) [20] (S3 Figure). If Mca could cleave GlcNAc to form GlcN, there would be fluorescence produced in the reaction mixture. Just as expected, fluorescence production was observed for GlcNAc treated with Mca and the observed rate of amide bond hydrolysis was 0.87±0.4 µM min^−1^ (Fig. 3B) measured with the GlcN standard curve (Fig. 3A). To determine steady-state kinetic parameters, deacetylase enzyme activity was measured with 0–5.0 mM GlcNAc. Michaelis-Menten equation was fitted to the data, yielding the following parameter values for GlcNAc: *K*_m_ = 275.30±12 mM, *k*_cat_ = 3.90±0.7 min^−1^ and *k*_cat_/*K*_m_ = 0.21±0.3 M^−1 ^s^−1^ (Table 5). The observed *K*_m_ value for Mca (275.30 mM) was remarkably higher (7.24-fold) than that of MshB (38 mM) [20]. Additionally, there was a great change in the *k*_cat_ value for Mca (3.90±0.7 min^−1^) *vs* MshB (46±2.2 min^−1^) [20]. These results indicate that GlcNAc has certain affinity for Mca while Mca shows limited deacetylase activity. Similarly, values of *K*_m_ and *k*_cat_ for Mca amidase activity were obtained with Eadie-Hofstee plots using the FSA-based assay with MSmB as substrate (Table 5): *K*_m_ = 92.30±1.5 mM, *k*_cat_ = 3.56±0.9 s^−1^ and *k*_cat_/*K*_m_ = 38.51±3.6 M^−1 ^s^−1^, similar to that calculated from the kinetic parameters reported for *M. smegmatis* Mca [7]. These results demonstrate that although *C. glutamicum* Mca has certain deacetylase activity to GlcNAc, it is significantly lower than the amidase activity with even the least reactive MSH *S*-conjugate substrate (Table 5). That is, *C. glutamicum* Mca primarily exhibits amidase activity toward MSH *S*-conjugates.

**Table 5:** Michaelis-Menten parameters of Mca for *N*-deacetylation of GlcNAc and for amidase activity of MSmB.

Substrate	*K*_m_ (mM)	*K*_cat_ (S^−1^)	*K*_cat_/*K*_m_ (M^−1 ^S^−1^)
MSmB^a^	92.30±1.5	3.56±0.9	38.51±9.6
GlcNAc^b^	275.30±12	0.057±0.7	0.21±0.3

a Assays were performed using 10 µM enzyme and 0–10 mM MSmB in 50 mM HEPES (pH 7.5) at 37°C.

b Assays were performed in the presence of 10 µM enzyme and 0–5.0 mM GlcNAc in 50 mM HEPES (pH 7.5) at 30°C.

### The effect of metal ions on Mca activity

**Figure 4:**
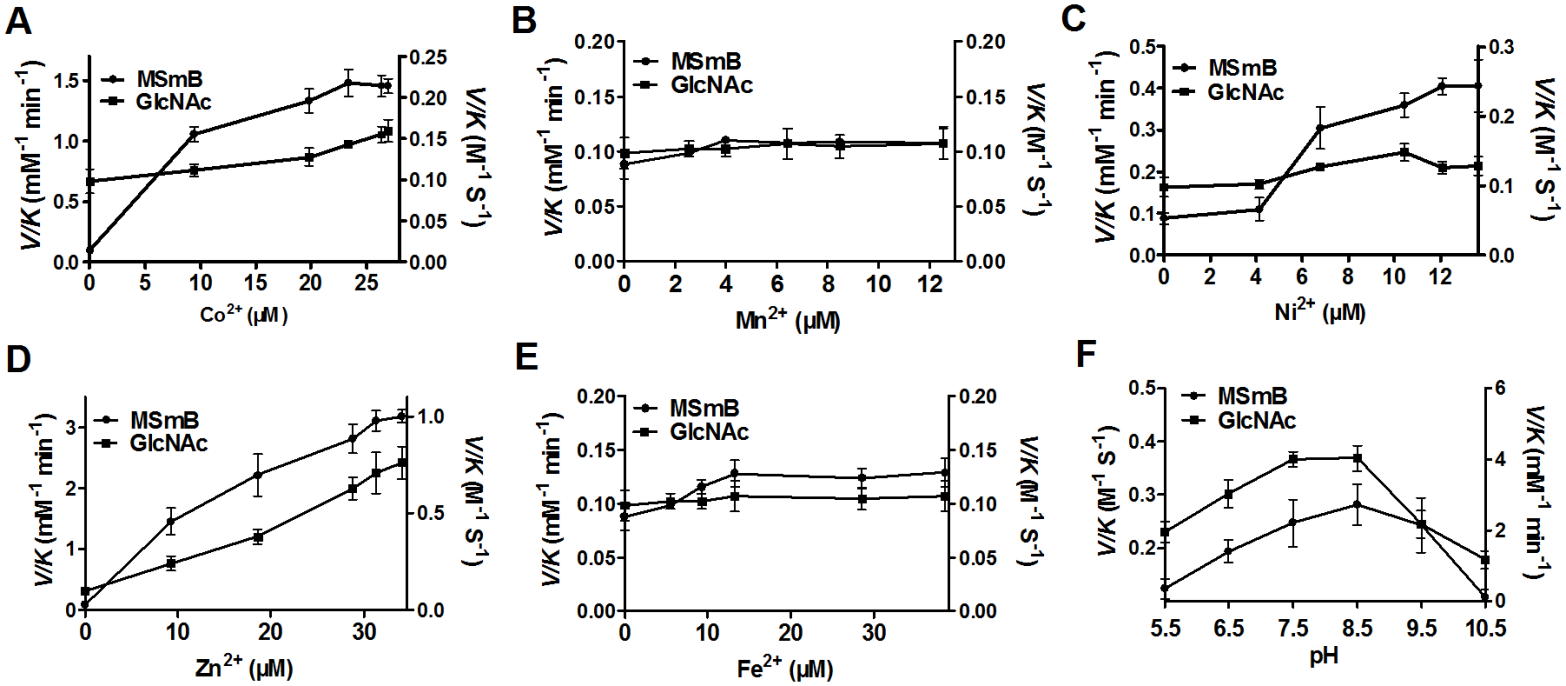
Effects of divalent metal cations and pH on *C. glutamicum* Mca activity. **A–E.** Catalytic activity of Mca in the presence of Co^2+^(A), Mn^2+^(B), Ni^2+^(C), Zn^2+^(D) and Fe^2+^(E), respectively, was analyzed with GlcNAc or MSmB as substrates. Apo-Mca was incubated with stoichiometric amounts of metal ions. After 30 min, the enzyme was diluted into assay buffer containing the substrate GlcNAc (5 mM) or MSmB (1 mM). The amidase activity (Left Y axis) and deacetylase activity (Right Y axis) were measured as described in “Materials and Methods”. **F.** Deacetylation of GlcNAc and amidase activity of MSmB by Zn^2+^-Mca at different pH levels. The *V/K* values were measured with 5 mM GlcNAc as substrate for deacetylase activity (Left Y axis) or 1 mM MSmB as substrate for amidase activity (Right Y axis) under six different pH values. *pK*_a_ values of 6.5 and 9.5 were determined by fitting Equation 1 to the data (bars represent standard error of the mean).

*C. glutamicum* Mca, similar to a metalloenzyme Mca in *M. smegmatis*, was shown to undergo reversible inhibition when treated with 1,10-phenanthroline, indicating that *C. glutamicum* Mca may be a metalloenzyme [27]. However, determination of what metal ions could restore and activate the activity of apo-Mca was not examined. Thus, the relative ability of various metal ions to activate Mca was measured. In the experiment, apo-Mca was reconstituted with a variety of divalent metal cations, and the initial rates of product formation were measured at different substrate concentrations. Interestingly, we observed that Mca exhibited the highest deacetylase and amidase activities with Zn^2+^ (Fig. 4D) and was moderately activated upon stoichiometric addition of Ni^2+^ (Fig. 4C) and Co^2+^ (Fig. 4A). However, no deacetylase and amidase activities of Mca were detected in the presence of Mn^2+^ (Fig. 4B) and Fe^2+^ (Fig. 4E). These results indicate that Mca is a metalloprotein with Zn^2+^ as metal ion cofactor.

### pH dependence of Mca-catalyzed reaction

The Mca-catalyzed reaction exhibited a bell-shaped dependence on pH (Fig. 4F), indicating that there are two ionizations of importance to the maximal deacetylase and amidase catalytic activity of Zn^2+^-Mca with the observed *pK*_a_ values of 6.5 and 9.5, respectively. The optimum pH for deacetylase and amidase activity of *C.glutamicum* Mca activity was between 7.5 and 8.5, similar to that of *M. tuberculosis* MshB [22].

### Mca activity affected by site-directed mutagenesis

Amino acid sequence alignments showed that *C. glutamicum* Mca shared conserved residues with other reference MshB and Mca proteins (S2 Figure). These conserved residues include Asp14, Asp15, Asp132, Asp141, Glu16, Glu43, Tyr137, His10, His12, His139 and His142. To date, whether mutation of these sites affects Mca activity has not been elucidated. To this end, a combination of mutagenesis and kinetic experiments was carried out. All mutant proteins purified by the Ni-NTA His⋅Bind Resin are as stable as the wild type Mca protein (S4 Figure). Mutation of Tyr137 and His139 led to an overall decrease in amidase and deacetylase activities of Mca (Tables 6&7), suggesting that the two residues are essential for catalytic activity of Mca in *C. glutamicum*. To both amidase and deacetylase, Asp14 and Asp141 mutations led to a modest increase (about 1.4–1.5 times) in *K*_m_, a 0.61–0.77 times decrease in *k*_cat_ and approximately 0.45-fold decrease in *k*_cat_/*K*_m_. Although *K*_m_ and *k*_cat_ of both amidase and deacetylase increased in the H142A mutant, their *k*_cat_/*K*_m_ slightly decreased (Tables 6&7). However, there was no effect of Asp15, Asp132, Glu16, Glu43, His10 and His12 on amidase and deacetylase activities. These results indicate that Asp14, Tyr137, His139 and Asp141 are important for Mca activity.

**Table 6:** Steady-state kinetic parameters of *C. glutamicum* Mca mutants for amidase activity of mycothiol bimane (MSmB).

Mca mutants	*K*_m_ (mM)	*k*_cat_ (min^−1^)	*k*_cat_/*K*_m_ (M^−1 ^s^−1^)	% WT activity
WT	92.34±1.5	3.56±0.9	38.5±1.6	100
10A	108.36±5.7	4.10±1.8	37.90	98.41
12A	111.62±2.2	3.97±1.5	35.80	93.03
14A	132.33±1.5	2.16±0.7	16.30	42.32
15A	128.76±1.5	4.68±1.4	36.30	94.35
16A	107.51±4.1	4.19±2.1	38.90	101.05
43A	93.37±1.5	3.46±3.9	37.10	96.47
132A	99.92±1.9	3.88±2.6	38.80	100.81
137A	542.37±3.3	0.30±0.9	0.560	1.53
139A	469.65±1.4	0.29±0.4	0.60	1.66
141A	137.97±1.8	2.74±2.3	19.90	51.74
142A	115.33±5.7	3.93±3.8	34.11±1.7	88.66

Assays were performed using 10 µM enzyme and 0–10 mM MSmB in 50 mM HEPES (pH 7.5) at 37°C.

**Table 7:** Steady-state kinetic parameters of *C. glutamicum* Mca mutants for *N*-deacetylation of *N*-acetyl-D-glucosamine (GlcNAc).

Mca mutants	*K*_m_ (mM)	*k*_cat_ (min^−1^)	*k*_cat_/*K*_m_ (M^−1 ^s^−1^)	% WT activity
WT	275.32±12	3.397±0.7	0.21±0.3	100
10A	275.34	3.40	0.21	97.91
12A	429.46	3.93	0.15	72.63
14A	338.77	2.37	0.12	55.51
15A	392.35	5.15	0.22	104.20
16A	299.90	3.57	0.19	94.57
43A	342.31	4.62	0.23	107.13
132A	307.65	3.59	0.19	92.64
137A	469.63	1.58	0.06	26.78
139A	407.96	2.04	0.08	39.71
141A	347.44	2.93	0.14	66.90
142A	318.37	3.78	0.18	94.32

Assays were performed in the presence of 10 µM enzyme and 0–5.0 mM GlcNAc in 50 mM HEPES (pH 7.5) at 30°C.

### Toxins-induced *mca* expression and its positive regulation by SigH

**Figure 5:**
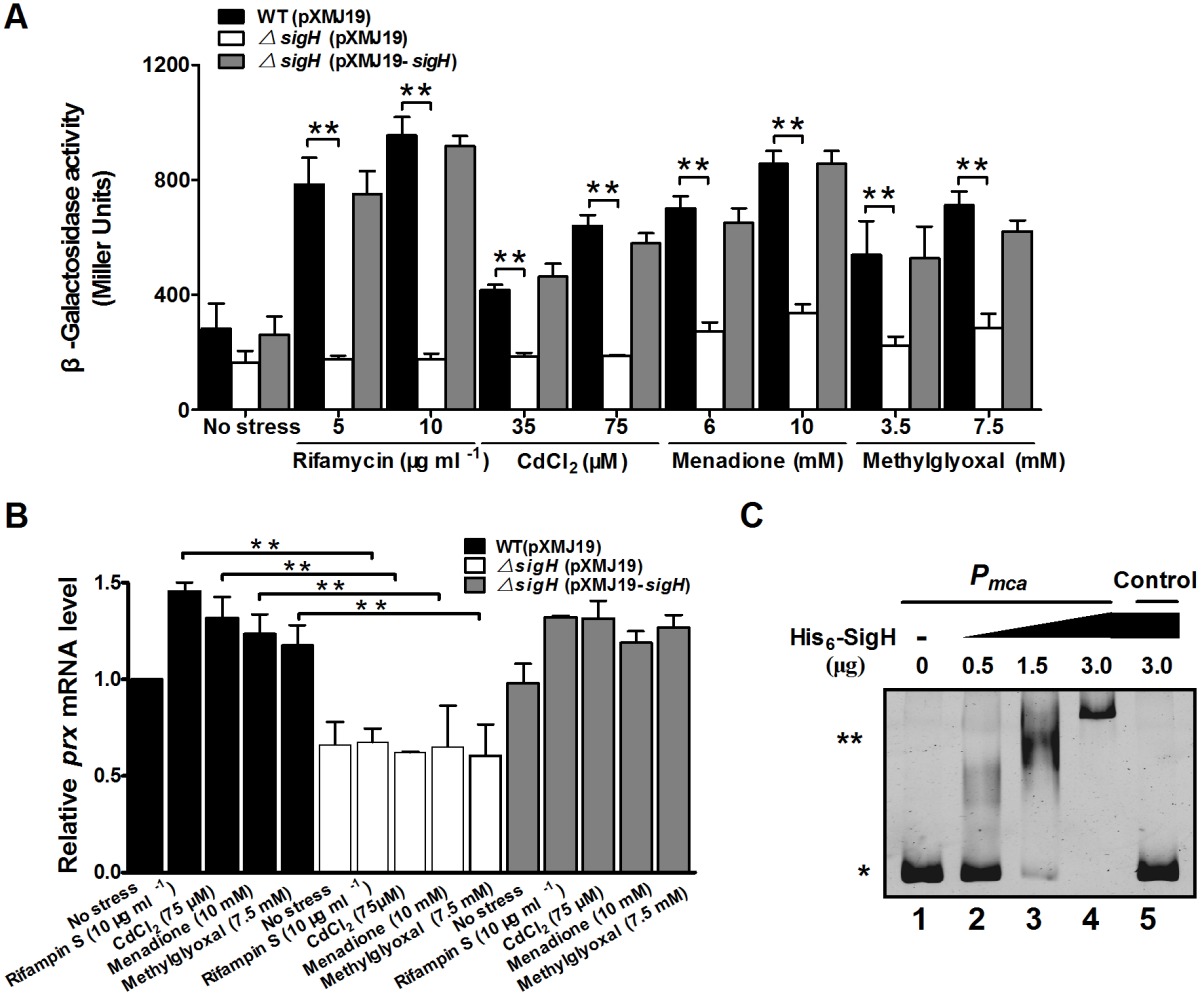
Positive regulation of *C. glutamicum mca* expression by SigH. **A.** β-Galactosidase analysis of the *mca* promoter activity was performed using the transcriptional *P_mca_::lacZ* chromosomal fusion reporter expressed in the wild type, *ΔsigH* mutant, and the complementary strain *ΔsigH*(pXMJ19-*sigH*). 100 µl of exponentially growing *C. glutamicum* cells induced with different toxic agents at indicated concentrations for 30 min was added to the enzyme reaction system. β-Galactosidase activity was assayed as described in “Materials and Methods”. Mean values with standard deviations (error bar) from at least three repeats are shown. **, *P≤*0.01. **B.** qRT-PCR assay revealed that expression of *mca* was under strict positive regulation of SigH. Exponentially growing *C. glutamicum* cells were exposed to different toxic agents at indicated concentrations for 30 min. The levels of *mca* expression were determined by quantitative RT-PCR. The mRNA levels were presented relative to the value obtained from wild type cells without treatment. The values represent the mean results from three independent cultivations, with standard errors. **: *P≤*0.01. **C.** Interactions between SigH and the *mca* promoter analyzed by EMSA. The increasing amounts of SigH used were 0, 0.5, 1.5, and 3.0 µg (lane 1, 2, 3, and 4, respectively). As a negative control, a 400 bp fragment from the *mca* coding region amplified with primers Control-F and Control-R instead of the 400 bp *mca* promoter was incubated with 3.0 µg His_6_-SigH in the binding assay (lane 5). (*) Free DNA, and (**) major DNA-protein complex.

Since Mca has been shown to promote survival of *C. glutamicum* in the presence of various toxins, qRT-PCR and *lac*Z activity profiling were employed to examine whether *mca* expression responds to these toxic stress inducers at the transcriptional level. The *lac*Z activity of *P_mca_::lacZ* chromosomal promoter fusion reporter in the RES167 wild type strain was quantitatively measured in bacterial cells either untreated or treated with different toxic agents of various concentrations (Fig. 5A). Concentrations of toxic agents applied were able to reduce the growth rate but under sub-lethal concentrations (S5 Figure). The level of *mca* expression was increased by approximately 3.37-, 2.39-, 3.03-, and 2.51-fold in the RES167 reporter strain treated with 10 µg ml^−1^ rifamycin S, 75 µM CdCl_2_, 10 mM MD, and 7.5 mM MG, respectively, as compared to untreated samples (Fig. 5A). Further, expression of the *P_mca_*::*lac*Z fusion displayed a dose-dependent increase in response to these adverse environmental conditions (Fig. 5A). These results clearly demonstrate that environmental stress induces *mca* expression, which in turn directly contributes to tolerance of *C. glutamicum* to these stress conditions. A similar dose-dependent pattern of *mca* expression in response to toxins rifamycin S, CdCl_2_, MD and MG was also observed in qRT-PCR analysis (Fig. 5B).

As SigH, the stress-responsive extracytoplasmic function-sigma (ECF-σ) factor, was reported to respond to thiol-oxidative stress and regulate the expression of multiple resistance genes [34], [35], we examined whether *mca* expression was subjected to SigH regulation by measuring the transcription of chromosomal *P_mca_*::*lac*Z fusions. Significant decrease of *lac*Z activity was observed for exponentially grown *ΔsigH* mutant exposed to 10 µg ml^−1^ rifamycin S, 75 µM CdCl_2_, 10 mM MD, and 7.5 mM MG for 30 min, as compared to the wild type (Fig. 5A). The reduced *mca* expression in the *ΔsigH* mutant was fully recovered in the complementary strain *ΔsigH*(pXMJ19-*sigH*) either under toxic-inducible or non-inducible conditions (Fig. 5A). SigH-depdendent *mca* activation was also confirmed by qRT-PCR analysis (Fig. 5B). These data suggest that SigH positively regulates the expression of *mca*.

To further determine whether the *mca* gene is directly regulated by SigH, *in vitro* EMSA assay was performed by the interaction of SigH (His_6_-SigH) with the *mca* promoter region. Incubation of His_6_-SigH with *P_mca_*, a 400 bp PCR fragment amplified from the *mca* promoter, led to retarded mobility of the probe (Fig. 5C), indicating direct binding of this protein to the *mca* promoter. Furthermore, the DNA-protein complexes increased in response to more His_6_-SigH used in the reactions. A 400 bp control DNA amplified from the *mca* coding region did not show detectable SigH binding (Fig. 5C, lane 5). Collectively, these results indicate that SigH activates *mca* expression by directly binding to the *mca* promoter.

## Discussion

Mca is an important amidase involved in detoxification of the MSH *S*-conjugates formed by MSH reacting with exogenous substrates, such as alkylating agents, antibiotics, and oxidants [8]. During detoxification, the amidase activity of Mca is mainly the cleavage of an amide bond in MSH moiety of the conjugates to yield AcCysmB and GlcN-Ins [7]. We demonstrated here that *C. glutamicum* Mca is a zinc metalloprotein that has both deacetylase and amidase activities, though the deacetylation rate of GlcNAc-Ins is lower than the deamination rate of MSmB *in vitro*. Our findings also revealed that Mca in *C. glutamicum* plays important roles in detoxification of alkylating agents, oxidants, antibiotics and heavy metals. The physiological roles of Mca in resistance to multiple toxins were further supported by the induced expression of Mca in *C. glutamicum* under various stress conditions, directly under the control of the stress-responsive extracytoplasmic function-sigma (ECF-σ) factor SigH.

*C. glutamicum* Mca exhibits amidase activity toward MSmB *in vitro*, the best substrate known for Mca and having the highest amidase activity (Table 5). Consistent with these results, when WT(pXMJ19), *Δmca*(pXMJ19) and *Δmca*(pXMJ19-*mca*) strains were incubated on ice with mBBr, the conjugate MSmB was produced in all three strains. While most of the MSmB conjugate was cleaved to yield AcCysmB in the wild type and *Δmca*(pXMJ19-*mca*) complementary strain, the conjugate was hardly converted to AcCysmB in the *Δmca* mutant (Fig. 2). This *in vivo* result confirmed that *C. glutamicum* Mca has amidase activity of cleaving MSH *S*-conjugates to detoxify some exogenous toxins. Interestingly, *C. glutamicum* Mca was also found closely related to *M. tuberculosis* MshB (30% sequence identity) (S2 Figure). MshB possesses GlcNAc-Ins deacetylase activity and transfers the acetyl groups from GlcNAc-Ins to form GlcN-Ins, which is an intermediate in the biosynthesis of MSH pathway [36]. Indeed, *C. glutamicum* Mca exhibited deacetylase activity with GlcNAc-Ins (Table 5), albeit markedly weaker than the amidase activity. In fact, an *mshB* null mutant in *C. glutamicum*, which exhibited similar resistance to many toxic reagents as the wild type, still produces low level of MSH [16]. However, whether Mca is actually involved in MSH synthesis in *C. glutamicum* by acting as an MshB substitute needs to be investigated in the future.

In the present study, detoxification ability of *C. glutamicum* Mca to various antibiotics was assessed *in vivo* by growth inhibition zone and MIC assays. As expected, the *mca* mutant showed increased sensitivity to some antibiotics of different types, including vancomycin, tetracycline, ciprofloxacin, rifamycin S, streptomycin and neomycin (Tables 3&4), consistent with the finding in *Streptomyces coelicolor* [8]. Examination of the structure of antibiotics found that many antibiotics have aromatic rings, α, β-unsaturated ketones, epoxide rings, arene oxides and quinones chemical structure, which could conjugate with MSH as they do with GSH [9], [10]. Furthermore, mercapturic acids of antibiotics have been found in the broth of some antibiotic-producing actinomycetes [12]. Therefore, the results presented here indicate a possible mechanism that the above-mentioned antibiotics first react with MSH, and then their conjugates are detoxified through Mca amidase. However, the *mca* mutant was not sensitive to some other antibiotics, such as lincomycin, erythromycin, gentamycin, spectinomycin, and penicillin. The reasons for the *mca* mutant to lose the sensitivity to these antibiotics may be as follows: the formation of the mycothiol S-conjugate suffices to detoxify the compound and the accumulation of the S-conjugate within the cell have little or no adverse consequence, or other detoxification pathways may be involved.

Some oxidants (e.g. plumbagin, napthoquinone and MD) can also rapidly form conjugates with MSH and the mutants disrupted in the mycothiol biosynthetic pathway are more sensitive to plumbagin, napthoquinone, and MD than the control strains [11]. In this study, the disruption of the *mca* gene in *C*. *glutamicum* resulted in significant increase in its sensitivity to MD. However, the *mca* mutant was not sensitive to low concentration of H_2_O_2_, CHP or diamide, and only slightly sensitive to very high concentration of H_2_O_2_, CHP, and diamide (Table 2), despite the sensitivity to either low or high concentrations of H_2_O_2_, CHP and diamide exhibited by mutants lacking MSH [16]. Thus, oxidants that can form stable *S-*conjugates (e.g., MD) may be cleaved by Mca to form mercapturic acids that are exported, while the low concentrations of oxidants (H_2_O_2_, CHP and diamide) acting as redox cycling agents may be mainly cleaned via other antioxidant pathway, and Mca may replenish other antioxidant pathway to work together only when cells are exposed to the high concentrations of peroxide, in line with the results of Steffek et al. [27].

Alkylating agents, including mBBr, IAM, NEM, CDNB and MG, having the maleimide ring structures and directly conjugating with MSH, had been reported previously [11]. In *M. tuberculosis* and *M. smegmatis*, a MSH-dependent detoxification pathway has been described, in which the MSH-NEM, MSH-mBBr, and MSH-IAM adducts produced in the cell serve as substrates for an amidase. The amidase catalyzes hydrolytic cleavage of the amide bond and converts these MSH *S*-conjugates to maleamic acid that is secreted into the medium [11]. Here, the *C. glutamicum mca* mutant was more susceptible to alkylating agents (including mBBr, IAM, NEM, CDNB and MG; Table 1) than the wild type, in line with the results of Rawat et al. reported for *Mycobacterium smegmatis* Mca [11]. Because a number of electrophilic compounds also have the maleimide ring structures, the MSH-dependent Mca detoxification pathway may protect the cell against electrophilic assault from such alkylating agents.

Important information arising from this study was that Mca is required to protect cells against heavy metals, including Cd^2+^, Cr^2+^, Ni^2+^ and Cu^2+^. Metal ions, such as Cd(II) and As(V), can form conjugates with GSH and MSH of thiols [37], [38]. These findings indicate that some heavy metal ions could form MSH *S*-conjugates that are cleaved by Mca to be detoxified. However, many other heavy metals, including Cd(II), have been shown to promote ROS production and subsequently lead to the elevation of intracellular GSSG, lower the GSH redox ratio [39]. At this time, Mca may also perform alternative mechanism to resist heavy metals-induced oxidative stress, *i.e.* Mca compensates MSH loss by playing deacetylase activity to make MSH-dependent peroxiredoxins function better.

In conclusion, this study showed that Mca, a SigH-dependent zinc metalloprotein that has both deacetylase and amidase activities, plays important roles in the detoxification of various alkylating agents, oxidants, antibiotics and heavy metals in *C. glutamicum*. Our insights into the versatile protective roles of Mca in *C. glutamicum* could be applied to enhance the robustness of this scientifically and commercially important bacterium in the future.

## Supporting Information

S1 Figure **Molecular weight determination of purified Mca.** **A.** SDS-PAGE analysis of proteins expressed in *E. coli* containing pET28a-*mca* plasmid. M, broad-range protein marker; lane 1, crude extract (5 µg) without IPTG induction; lane 2, crude extract (5 µg) with induction; lane 3, purified His_6_-Mca protein (5 µg). **B.** Molecular weight standard curve. **C.** Analysis of purified Mca by gel-filtration chromatography.
(TIF)

S2 Figure **Multiple sequence alignment of** ***C. glutamicum*** **Mca with other representative Mca and MsrB proteins.** Accession numbers: Mca from *C. glutamicum* (NP_600215), *Catenulispora acidiphila* (YP_003111447), *Beutenbergia cavernae* (YP_002881101), *Nocardiopsis dassonvillei* (YP_003678053), *Stackebrandtia nassauensis* (YP_003509529), *Streptomyces* sp. (YP_007861426), *Tsukamurella paurometabola* DSM 20162 (YP_003648071), *Rhodococcus qingshengii* (WP_007726383), *Mycobacterium tuberculosis* (NP_215598), *Streptomyces coelicolor* A3(2) (NP_629119), *Verrucosispora maris* (WP_013731662); MshB from *Mycobacterium tuberculosis* (NP_215686), *Mycobacterium smegmatis* (WP_003896526), *Rhodococcus equi* (WP_022594822) and *Streptomyces davawensis* JCM 4913 (YP_007521786).
(TIF)

S3 Figure **Fluorescence measured following reaction of glucosamine (GlcN) and** ***N*** **-acetyl-D-glucosamine (GlcNAc) with FSA.** Solutions of GlcNAc (5 mM) in buffer (50 mM Hepes, 50 mM NaCl, 1 mM tris(2-carboxyethyl)phosphine, pH7.5) were diluted with borate (pH9.0) and then incubated with FSA (final concentration 2.3 mM), and the resulting fluorescence was measured (excitation 395 nm, emission 485 nm).
(TIF)

S4 Figure **SDS-PAGE analysis of purified Mca proteins.** M: protein molecular weight marker; Lane 1: Mca wild type; Lanes 2–12: Mca H10A, H12A, D14A, D15A, E16A, E43A, D132A, Y137A, H139A, D141A and H142A mutants, respectively.
(TIF)

S5 Figure **Growth curves of** ***C. glutamicum*** **in response to sub-lethal concentrations of toxins.** *C. glutamicum* wild type was grown in LB medium to an *A_600_* of 0.6–0.7 and exposed to different toxic agents of various concentrations. The cultures continued to be incubated for 10 h, and the *A*_600_ was measured in 2 h intervals.
(TIF)

S1 Table **Bacterial strains, plasmids, and primers used in this study.**
(DOCX)
