# Supplementary figures and images for: Functional Characterization of Corynebacterium glutamicum Mycothiol S-Conjugate Amidase
Source: PLoS One. 2014 Dec 16;9(12):e115075. doi: 10.1371/journal.pone.0115075 (PMC4267739; doi:10.1371/journal.pone.0115075)

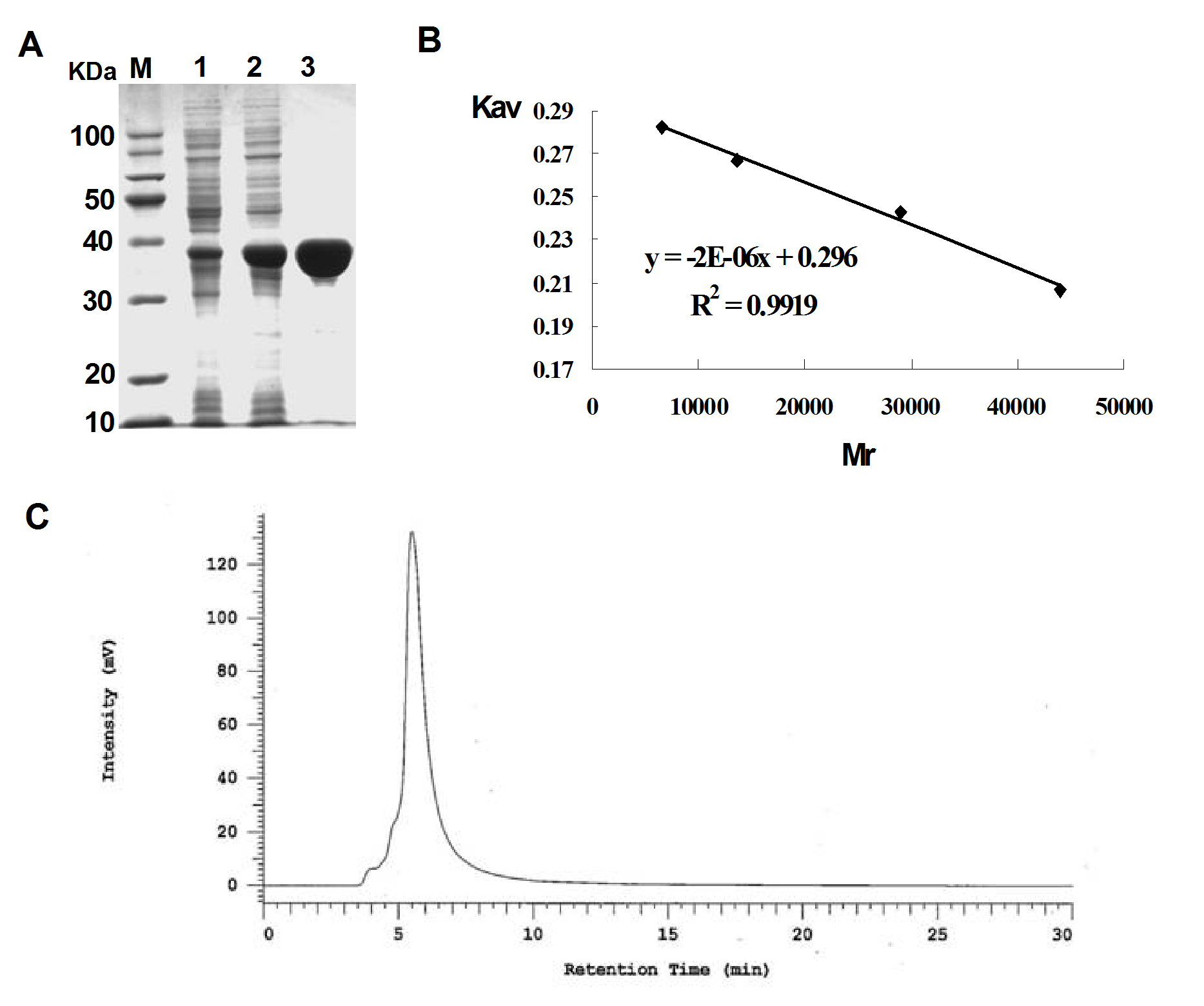

Supplement: S1 Figure — Molecular weight determination of purified Mca. A. SDS-PAGE analysis of proteins expressed in E. coli containing pET28a-mca plasmid. M, broad-range protein marker; lane 1, crude extract (5 µg) without IPTG induction; lane 2, crude extract (5 µg) with induction; lane 3, purified His6-Mca protein (5 µg). B. Molecular weight standard curve. C. Analysis of purified Mca by gel-filtration chromatography. (TIF) [file pone.0115075.s001.tif]

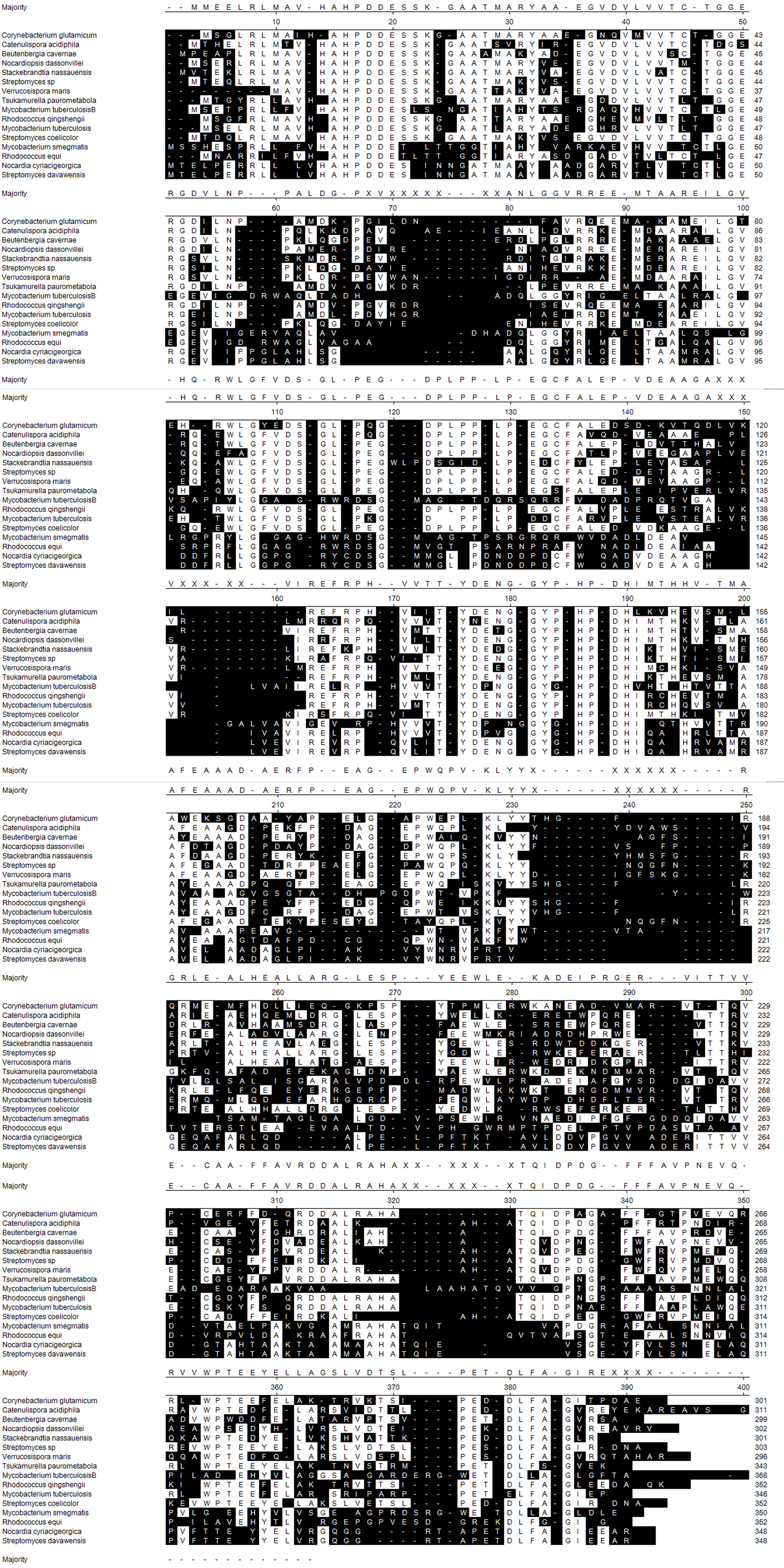

Supplement: S2 Figure — Multiple sequence alignment of C. glutamicum Mca with other representative Mca and MsrB proteins. Accession numbers: Mca from C. glutamicum (NP_600215), Catenulispora acidiphila (YP_003111447), Beutenbergia cavernae (YP_002881101), Nocardiopsis dassonvillei (YP_003678053), Stackebrandtia nassauensis (YP_003509529), Streptomyces sp. (YP_007861426), Tsukamurella paurometabola DSM 20162 (YP_003648071), Rhodococcus qingshengii (WP_007726383), Mycobacterium tuberculosis (NP_215598), Streptomyces coelicolor A3(2) (NP_629119), Verrucosispora maris (WP_013731662); MshB from Mycobacterium tuberculosis (NP_215686), Mycobacterium smegmatis (WP_003896526), Rhodococcus equi (WP_022594822) and Streptomyces davawensis JCM 4913 (YP_007521786). (TIF) [file pone.0115075.s002.tif]

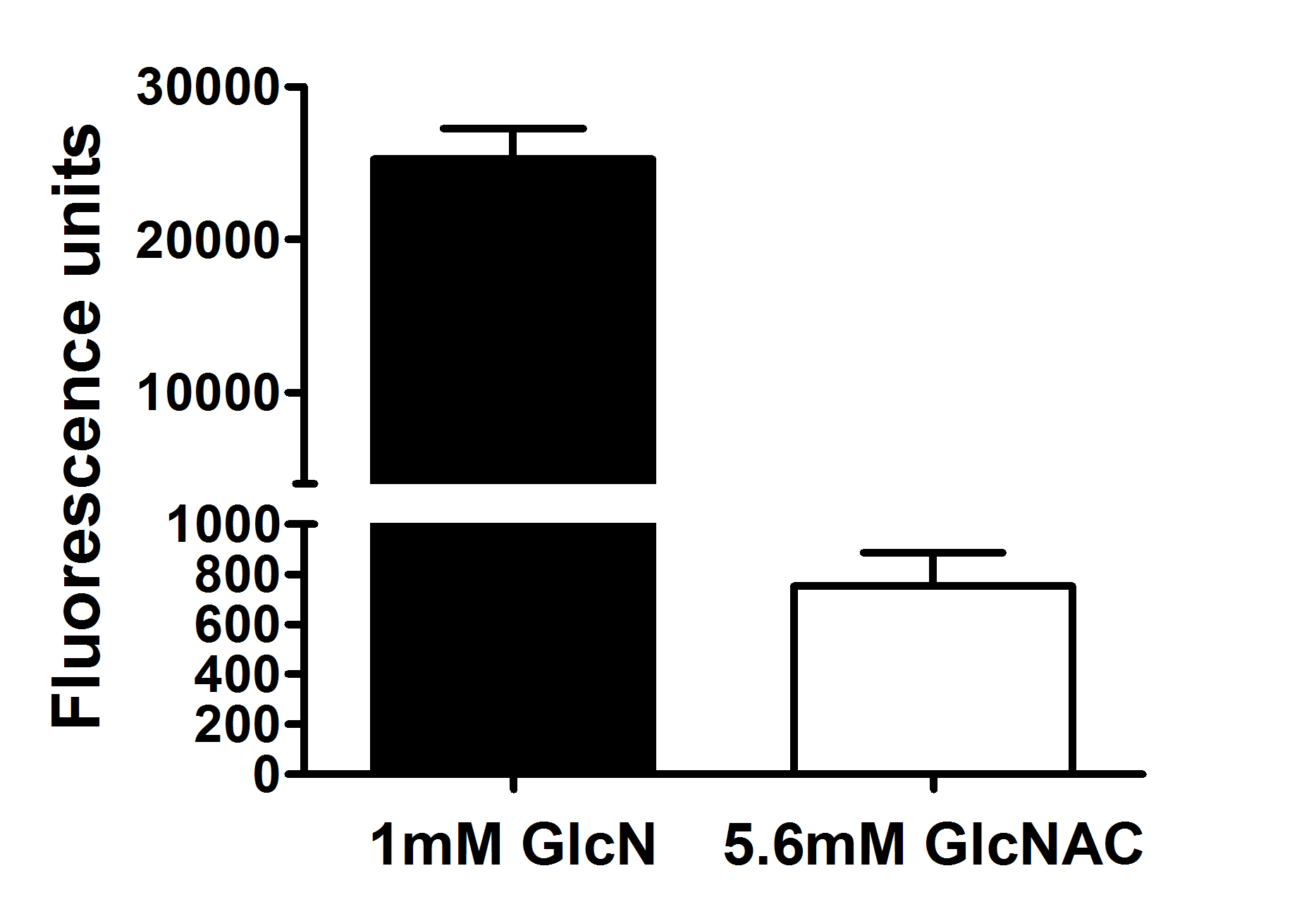

Supplement: S3 Figure — Fluorescence measured following reaction of glucosamine (GlcN) and N -acetyl-D-glucosamine (GlcNAc) with FSA. Solutions of GlcNAc (5 mM) in buffer (50 mM Hepes, 50 mM NaCl, 1 mM tris(2-carboxyethyl)phosphine, pH7.5) were diluted with borate (pH9.0) and then incubated with FSA (final concentration 2.3 mM), and the resulting fluorescence was measured (excitation 395 nm, emission 485 nm). (TIF) [file pone.0115075.s003.tif]

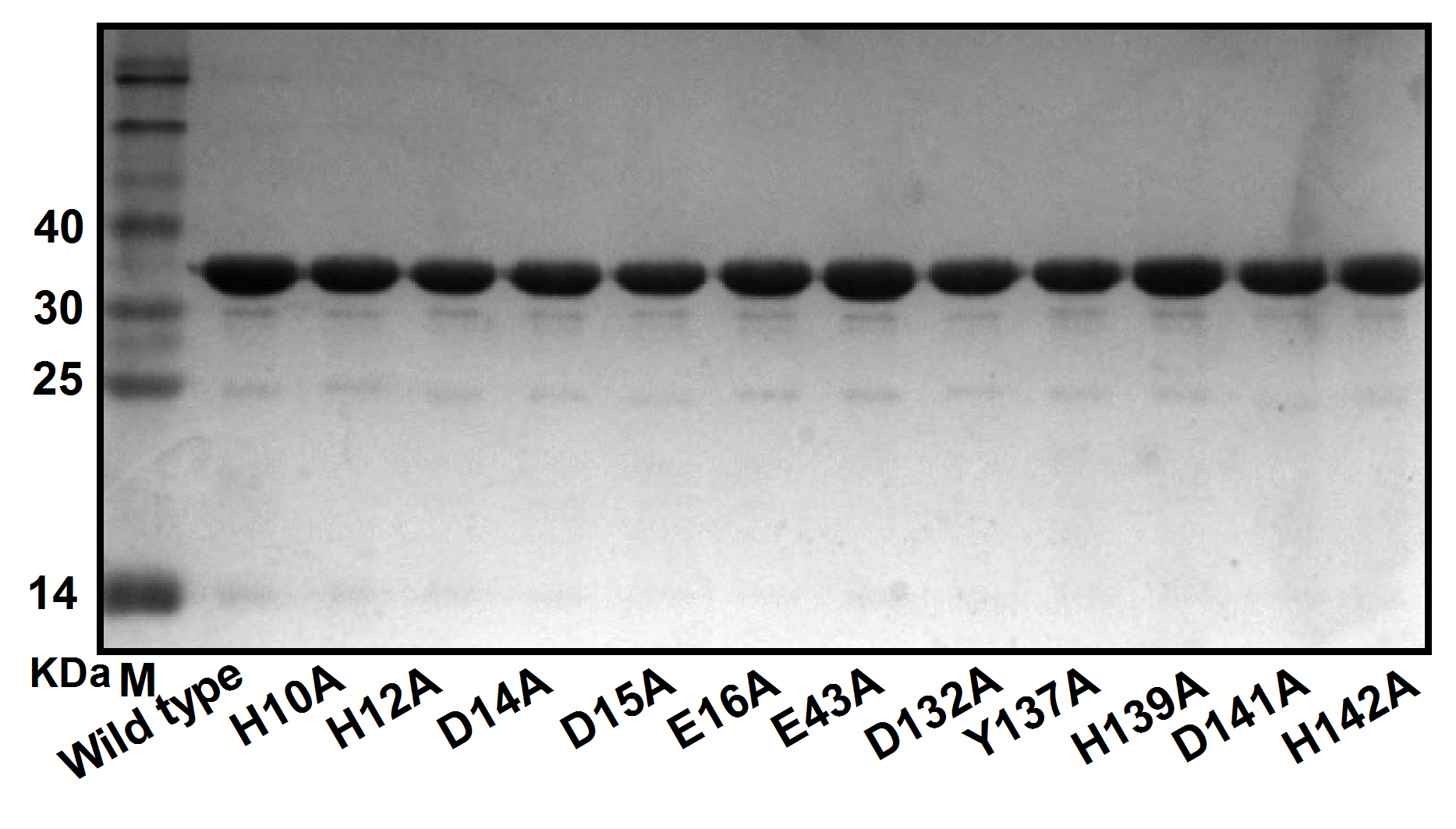

Supplement: S4 Figure — SDS-PAGE analysis of purified Mca proteins. M: protein molecular weight marker; Lane 1: Mca wild type; Lanes 2–12: Mca H10A, H12A, D14A, D15A, E16A, E43A, D132A, Y137A, H139A, D141A and H142A mutants, respectively. (TIF) [file pone.0115075.s004.tif]

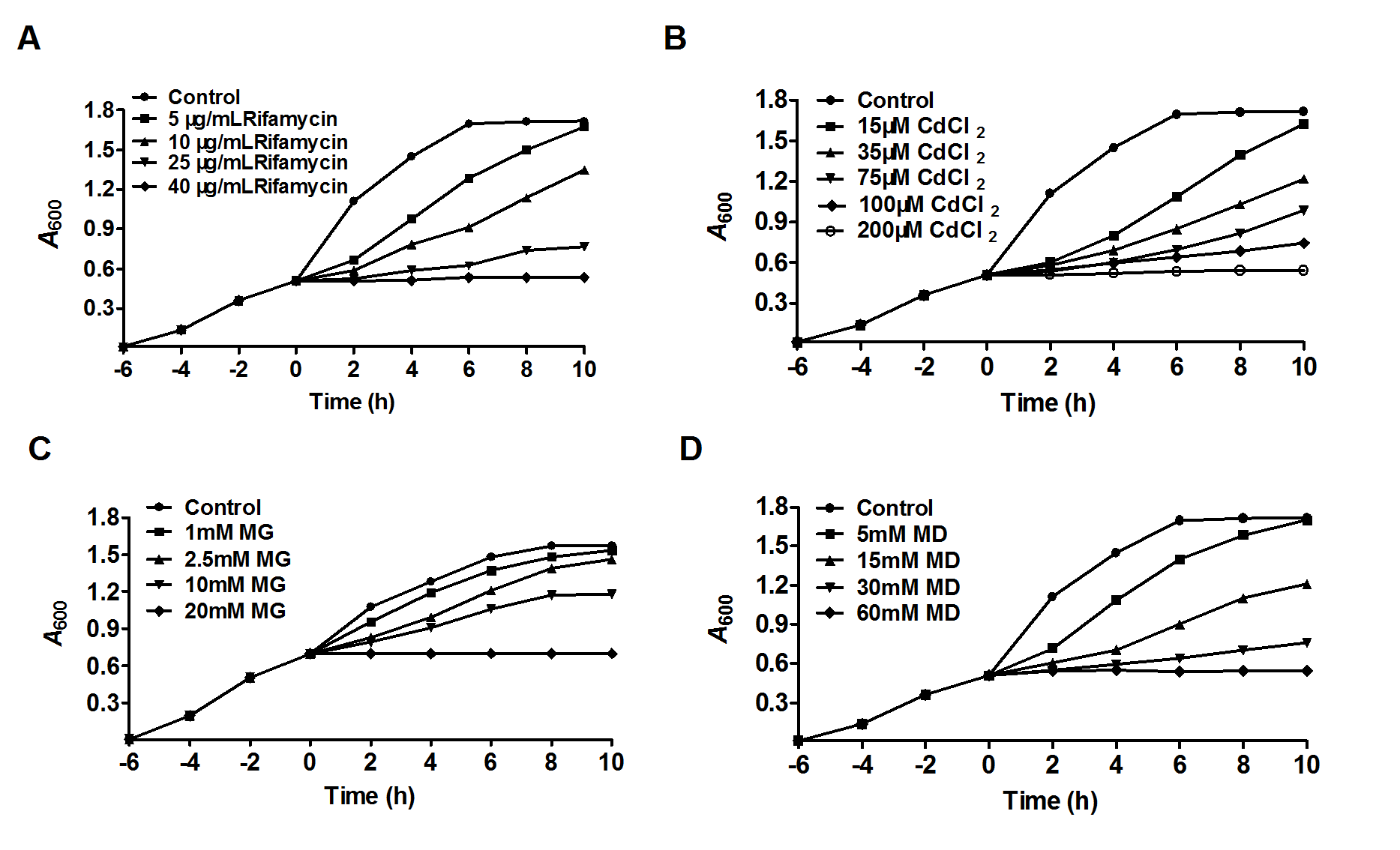

Supplement: S5 Figure — Growth curves of C. glutamicum in response to sub-lethal concentrations of toxins. C. glutamicum wild type was grown in LB medium to an A600 of 0.6–0.7 and exposed to different toxic agents of various concentrations. The cultures continued to be incubated for 10 h, and the A 600 was measured in 2 h intervals. (TIF) [file pone.0115075.s005.tif]
