# Supplementary material for: Functional Characterization of Corynebacterium glutamicum Mycothiol S-Conjugate Amidase
Source: PLoS One. 2014 Dec 16;9(12):e115075. doi: 10.1371/journal.pone.0115075 (PMC4267739; doi:10.1371/journal.pone.0115075)
Supplement: S1 Table — Bacterial strains, plasmids, and primers used in this study. (DOCX) [file pone.0115075.s006.docx]

**Table S1. Bacterial strains, plasmids, and primers used in this study**

| **Strains, plasmids, and primers** | **Relevant characteristics or sequence** | **Source or reference** |
| --- | --- | --- |
| ***E. coli*** |  |  |
| JM109 | *recA1 supE44 endA1 hsdR17 gyrA96 relA1 thi Δ*(*lac-proAB*) F′(*traD36proAB lacI*^q^ *lacΔZM15*) | Stratagene |
| BL21(DE3) | *E. coli* expression host, *hsdS gal* (*λc*I*ts*857 *ind-l* *Sam7 nin-*5 *lac UV5-*T7 gene 1) | Novagen |
| ***C. glutamicum*** |  |  |
| RES167 | Restriction-deficient mutant of ATCC13032, (*cglIM-cglIR-cglIIR*) | Tauch et al. 2002 |
| RES167Δ*mca* | *mca* deleted in RES167 | This study |
| RES167Δ*sigH* | *sigH* deleted in RES167 | This study |
| WT(pXMJ19) | RES167 containing pXMJ19 vector | This study |
| *Δmca*(pXMJ19) | *Δmca* containing pXMJ19 vector | This study |
| *ΔsigH*(pXMJ19) | *ΔsigH* containing pXMJ19 vector | This study |
| *Δmca*(pXMJ19-*mca*) | Complementation of *mca* in *Δmca* | This study |
| *ΔsigH*(pXMJ19-*sigH*) | Complementation of *sigH* in *ΔsigH* | This study |
| **Plasmids** |  |  |
| pXMJ19 | Shuttle vector (*P_tac_ lacI^q^ pBL1 oriV_C. glutamicum_* pK18 *oriV_E. coli_*) | Jakoby et al. 1999 |
| pXMJ19-*mca* | *mca* fragment inserted into pXMJ19 | This study |
| pXMJ19-*sigH* | *sigH* cloned into pXMJ19 for complementation | This study |
| pK18*mobsac*B | Suicide plasmid carrying *sacB* for selecting double crossover in *C. glutamicum*, Km^r^ | Schäfer et al. 1994 |
| pK18*mobsacBΔmca* | Carrying *mca* deletion | This study |
| pK18*mobsacBΔsigH* | Construct used for in-frame deletion of *sigH* | This study |
| pK18*mobsacB-P_mca_::lacZ* | *lacZ* fusion reporter vector | This study |
| pET28a(+) | Expression vector with *N*-terminal hexahistidine affinity tag | Novagen |
| pET28a-*mca* | pET28a derivative for expression of *mca* | This study |
| pET28a-*mca:H10A* | pET28a derivative for expression of *mca: H10A* | This study |
| pET28a-*mca:H12A* | pET28a derivative for expression of *mca:H12A* | This study |
| pET28a-*mca:D14A* | pET28a derivative for expression of *mca:D14A* | This study |
| pET28a-*mca:D15A* | pET28a derivative for expression of *mca:D15A* | This study |
| pET28a-*mca:E16A* | pET28a derivative for expression of *mca:E16A* | This study |
| pET28a-*mca:E43A* | pET28a derivative for expression of *mca:E43A* | This study |
| pET28a-*mca:D132A* | pET28a derivative for expression of *mca:D132A* | This study |
| pET28a-*mca:Y137A* | pET28a derivative for expression of *mca:Y137A* | This study |
| pET28a-*mca:H139A* | pET28a derivative for expression of *mca:H139A* | This study |
| pET28a-*mca:D141A* | pET28a derivative for expression of *mca:D141A* | This study |
| pET28a-*mca:H 142A* | pET28a derivative for expression of *mca:H 142A* | This study |
| **Primers** |  |  |
| DMcaF1 | CAAGGATCCAAAGGAGGACAACCGTGAGTGGTCTACGCC (BamHI) | To generate pK18*mobsacBΔmca* |
| DMcaR1 | CAAGAATTCTTATTCTGCATCGGGTG |  |
| DMcaF2 | CAAGGATCCGGAAACTGCTGGTTCGCGGG |  |
| DMca-R2 | CCAGTCGACTGTCAGTGGGTTTGCGGCG (SalI) |  |
| EMcaF | CGCGGATCCGTGAGTGGTCTACGCCTAATGG (BamHI) | To generate pET28a-*mca* |
| EMcaR | ACGCGTCGACTTATTCTGCATCGGGTGTGATTC (SalI) |  |
| CMcaF | CAAGGATCCAAAGGAGGACAACCGTGAGTGGTCTACGCC (BamHI) | To generate pXMJ19-*mca* |
| CMcaR | CAAGAATTCTTATTCT*GC*ATCGGGTG (EcoRI) |  |
| McaH10AF | GCCTAATGGCGATC*GC*CGCCCACCCTGACG | For site-directed mutagenesis H10A |
| McaH10AR | CGTCAGGGTGGGCGG*CG*ATCGCCATTAGGC |  |
| McaH12AF | TGGCGATCCACGCC*GC*CCCTGACGACGAGT | For site-directed mutagenesis H12A |
| McaH12AR | ACTCGTCGTCAGGG*GC*GGCGTGGATCGCCA |  |
| McaD14AF | CCACGCCCACCCTTGCCGACGAGTCAAGCA | For site-directed mutagenesis D14A |
| McaD14AR | TGCTTGACTCGTCGGCAAGGGTGGGCGTGG |  |
| McaD15AF | CCCACCCTGACG*C*CGAGTCAAGCAA | For site-directed mutagenesis D15A |
| McaD15AR | TTGCTTGACTCG*G*CGTCAGGGTGGG |  |
| McaE16AF | ACCCTGACGACG*C*GTCAAGCAAGGG | For site-directed mutagenesis E16A |
| McaE16AR | CCCTTGCTTGAC*G*CGTCGTCAGGGT |  |
| McaE43AF | GCACTGGTGGTG*C*GCGTGGAGACAT | For site-directed mutagenesis E43A |
| McaE43AR | ATGTCTCCACGC*G*CACCACCAGTGC |  |
| McaD132AF | CATCATTACCTATG*C*TGAGAACGGCGGTT | For site-directed mutagenesis D132A |
| McaD132AR | AACCGCCGTTCTCA*G*CATAGGTAATGATG |  |
| McaY137AF | GATGAGAACGGCGGT*GC*CCCACACCCGGATCA | For site-directed mutagenesisY137A |
| McaY137AR | TGATCCGGGTGTGGG*GC*ACCGCCGTTCTCATC |  |
| McaH139AF | GAACGGCGGTTACCC*GG*ACCCGGATCACCT | For site-directed mutagenesis H139A |
| McaH139AR | AGGTGATCCGGGT*CC*GGGTAACCGCCGTTC |  |
| McaD141AF | TTACCCACACCCG*GC*TCACCTCAAGGTTC | For site-directed mutagenesis D141A |
| McaD141AR | GAACCTTGAGGTGA*GC*CGGGTGTGGGTAA |  |
| McaH142AF | ACCCACACCCGGAT*GC*CCTCAAGGTTCATG | For site-directed mutagenesis H142A |
| McaH142AR | CATGAACCTTGAGG*GC*ATCCGGGTGTGGGT |  |
| *Pmca*-F1 | TCCCCCGGGGCAGGGCGGTGTGCGATG (SmaI) | To generate pK18*mobsacB-* *P_mca_::lacZY* |
| *Pmca*-R | TCTAGAGCGTAGACCACTCACGTGTTTC (Xbal) |  |
| *Pmca*-F2 | GGCGATCACCAGCAGCACGCC | To gain 400 bp *mca* promoter DNA segment |
| lacZY-F | GAAACACGTGAGTGGTCTACGCTCTAGAACTAGTATGACCATGATTACGGATTC (SpeI) | To generate *lacZY* fragment |
| lacZY-R | AAAACTGCAGTTAAGCGACTTCATTCACCTG (*Pst*I) |  |
| DsigH- F1 | TCCCCCGGGCGCGCATTTCTCGGGTTGGAG (*Sma*I) | To generate pK18*mobsacB-ΔsigH* |
| DsigH- R1 | CCGAATTCGTGTCGAGCATCGTGGCAGTGCCTCCTCTTC |  |
| DsigH- F2 | CCGAATTCGTGTCGAGCATC |  |
| DsigH- R2 | ACGCGTCGACCACCGGTGCAGCGCAAGATG (*Sal*I) |  |
| sigH-F | CGCGGATCCATGGCTGAAAACCGAACC (*BamH*I) | To generate pET28a-*sigH* and pXMJ19- *sigH* |
| sigH-R | ACGCGTCGACTTATGCCTCCGAATTTTTC (*Sal*I) |  |
| Control-F | GGATAAGCCAGGAATCCTGG | To generate control DNA for EMSA |
| Control-R | CTCATCATAGGTAATGATGAC |  |

Underlined sites indicate restriction enzyme cutting sites added for cloning. Letters in italics denote the mutation sites in overlap PCR for site-directed mutagenesis.

**References**

Tauch A, Kirchner O, Loffler B, Gotker S, Pühler A, et al. (2002) Efficient electrotransformation of *Corynebacterium diphtheriae* with a mini-replicon derived from the plasmid pGA1. Curr. Microbiol. 45: 362–367.

Jakoby M, Ngouoto-Nkili C E, Burkovski A (1999) Construction and application of new *Corynebacterium glutamicum* vectors. *Biotechnol* *Technique****s*** 13: 437–441.

Schäfer A, Tauch A, Jager W, Kalinowshi J, Thierbach G, et al. (1994) Small mobilizable multi-purpose cloning vectors derived from the *Escherichia coli* plasmids pK18 and pK19: selection of defined deletions in the chromosome of *Corynebacterium glutamicum*. *Gene* 145: 69–73.
